# Insertable Electrochemical Microsensors for In Vivo and In Situ Remote Monitoring of Plant Stress Biomarkers

**DOI:** 10.1021/acsmeasuresciau.6c00094

**Published:** 2026-08-20

**Authors:** Arezoo Saadati, Hatef Bokaei, Lukas Richtera, Jan Zouhar, Amir M. Ashrafi

**Affiliations:** † Central European Institute of Technology, Brno University of Technology, Purkyňova 123, CZ-612 00 Brno, Czech Republic; ‡ Department of Chemistry and Biochemistry, 309613 Mendel University, Zemedelska 1, CZ613 00 Brno, Czech Republic; § Central European Institute of Technology, Mendel University CZ61300, Brno, Czech Republic; ∥ 119450 Institute of Photonic and Electronics of the Czech Academy of Sciences, Chaberská 57, 18200 Prague 8, Czechia

**Keywords:** insertable microsensors, microneedle, plant stress biomarkers, in vivo and in situ measurement, remote monitoring, smart agriculture, reactive oxygen species, phytohormones

## Abstract

Plant productivity, a critical component of global food production, is significantly influenced by abiotic and biotic stress factors. Thus, timely identification of these stresses is crucial for preventing irreparable damage to plant health and productivity. Biomarkers, including ions, phytohormones, gaseous analytes, biomolecules, oxidative stress-related species, and pathogen deoxyribonucleic acid (DNA), can serve as indicators for monitoring the health status of plants. Therefore, continuous real-time monitoring of these biomarkers using sensitive methods with short response times is an attractive approach that enables timely action to address potential problems. Unlike conventional analytical techniques, insertable microsensors can be placed within plant tissue to monitor its physiological condition. This review highlights the application of insertable electrochemical microsensors for real-time assessment of plant stress-related biomarkers and the importance of remote monitoring in smart agriculture. Finally, current challenges and prospects for the widespread application of insertable microsensors are discussed.

## Graphical abstract

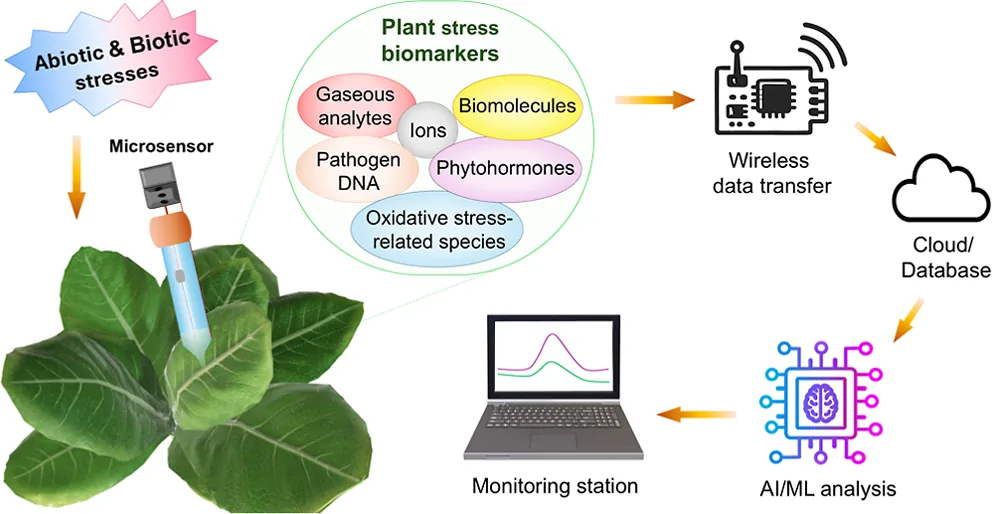

## 1 Introduction

Crop losses due to biotic and abiotic stresses pose a major challenge to global food security by significantly reducing agricultural productivity worldwide.^1,2^ Environmental factors such as drought, salinity, extreme temperatures, and pathogen infections affect plant growth rate and yield.^3^ Plant stress responses involve complex biochemical processes, including the generation of reactive oxygen species (ROS), hormonal signaling, and ionic fluctuations. These processes lead to the accumulation of specific metabolites and signaling molecules that act as reliable biomarkers for stress detection.^4,5^ Therefore, determining the accumulation of specific biomarkers can be crucial for the early diagnosis of abiotic and biotic stresses, timely intervention, and effective crop management.^4^

Various methods have been utilized to determine plant stress biomarkers. A bioassay, employed as a reporter system in combination with thin-layer chromatography or paper chromatography, was the first technique used to identify plant biomarkers, although it lacks specificity.^6^ Therefore, these techniques can be used in combination with high-performance thin-layer chromatography (HPTLC) or high-performance liquid chromatography (HPLC) to detect phytohormones.^6,7^ Endogenous auxin has been measured using immunological techniques such as immunocytochemistry and radioimmunoassay.^8,9^ However, these approaches are not widely used owing to their high antibody costs and inconsistent test results.^6^ In general, separation techniques, such as gas chromatography,^10^ HPLC,^11^ ultraperformance liquid chromatography (UPLC),^12^ and capillary electrophoresis,^13^ which require similar preparation steps, including sample collection, pretreatment, and extraction,^6,14^ are combined with detection and quantification methods, including mass spectrometry (MS)^14^ or tandem MS,^15^ fluorescence monitoring,^16^ spectrophotometry,^17^ UV monitoring,^18^ and enzyme-linked immunosorbent assay (ELISA).^19^ However, ELISA is susceptible to impurities and requires high-quality antibodies.^6^ Despite their robustness, high precision, and accuracy, these techniques rely on operator skill and reagent purity. Furthermore, these destructive methods damage the sample and require a long analysis time. Additionally, they need expensive and bulky equipment, unsuitable for online and remote monitoring of environmental stressors in plants in situ or in vivo.^20,21^ To address these shortcomings, researchers have focused on sensors and biosensors that combine a recognition module and a signal transduction component for the rapid monitoring of plant growth.^21,22^ The design of effective biointerface (associated with selection and preparation of materials) and signal transduction and amplification methods, which may be based on field-effect transistors (FETs), optical, or electrochemical methods, has received the majority of attention in reported studies.^22,23^ Electrochemistry is a convenient signal transduction technology because of its cost-effectiveness, simplicity, high sensitivity, short response time, and ease of integration.^6^ In addition, electrochemical methods are well suited for use in microscopic environments because they can be miniaturized. This enables the acquisition of chemical information with high spatial resolution, and measurements can be performed in tissues and living cells with minimal damage or perturbation. Advanced technologies, such as insertable electrochemical microsensors, have been proposed as a practical approach for plant health monitoring.^23^ The use of these technologies in agriculture can lead to better decisions regarding plant watering and fertilizing, thereby increasing productivity while reducing waste and environmental harm.

This review provides a comprehensive overview of insertable electrochemical microsensors in plants, focusing on their application for in vivo and in situ analysis and remote monitoring of stress-related biomarkers, and further discusses the analytical approaches employed for their accurate and quantitative determination.

Although several excellent reviews have recently summarized advances in plant sensing technologies, each has focused on a specific aspect. Coatsworth et al.^23^ discussed continuous monitoring in plants from a plant physiological perspective, emphasizing the biological significance of in vivo sensing rather than sensor engineering. Kumari et al.^24^ and Wang et al.^25^ focused on microneedle (MN)-based sensing technologies, including their fabrication, mechanical properties, and applications in agriculture. Similarly, recent reviews on wearable and implantable electrochemical sensors have highlighted flexible sensing platforms for monitoring plant metabolites and physiological processes.^26,27^ In contrast, the present review adopts a biomarker-centered perspective, correlating plant stress physiology with insertable electrochemical microsensors developed for the in vivo and in situ detection of ions, phytohormones, gaseous analytes, biomolecules, oxidative stress-related species, and pathogen-derived nucleic acids. Furthermore, beyond sensor development, it discusses the integration of insertable microsensors with wireless communication, the Internet of Things (IoT), artificial intelligence (AI), machine learning (ML), and remote monitoring systems to provide a comprehensive framework for translating electrochemical plant sensing technologies into practical applications in precision agriculture.

## 2 Application of Electrochemical Microsensors in Plants

Microelectrodes are miniaturized working electrodes with micrometer dimensions that have revolutionized in vivo chemical analysis by enabling precise and minimally invasive electroanalysis in living systems.^28–30^ Their small size allows them to be inserted into plant tissues, enabling real-time, spatially resolved monitoring of stress-related biomarkers with minimal disruption.^26^ Depending on the severity and duration of adverse environmental conditions, various stress responses are triggered, which can be identified by the biochemical and physiological changes they induce in plants.^31^ Microelectrodes have been employed to investigate processes such as leaf respiration and the release of plant compounds.^32,33^ In this review, microsensor applications are categorized into major classes of target analytes with distinct biological functions, including ions, phytohormones, gaseous analytes, biomolecules, oxidative stress-related species, and pathogen DNA. Figure 1 provides an overview of the main insertable microsensor types, functional materials, target biomarker classes, transduction pathways, and their integration into remote monitoring and smart agriculture systems. The works reporting the use of insertable microsensors for detecting stress-related biomarkers and for DNA extraction are summarized in Table 1.

**Figure 1:**
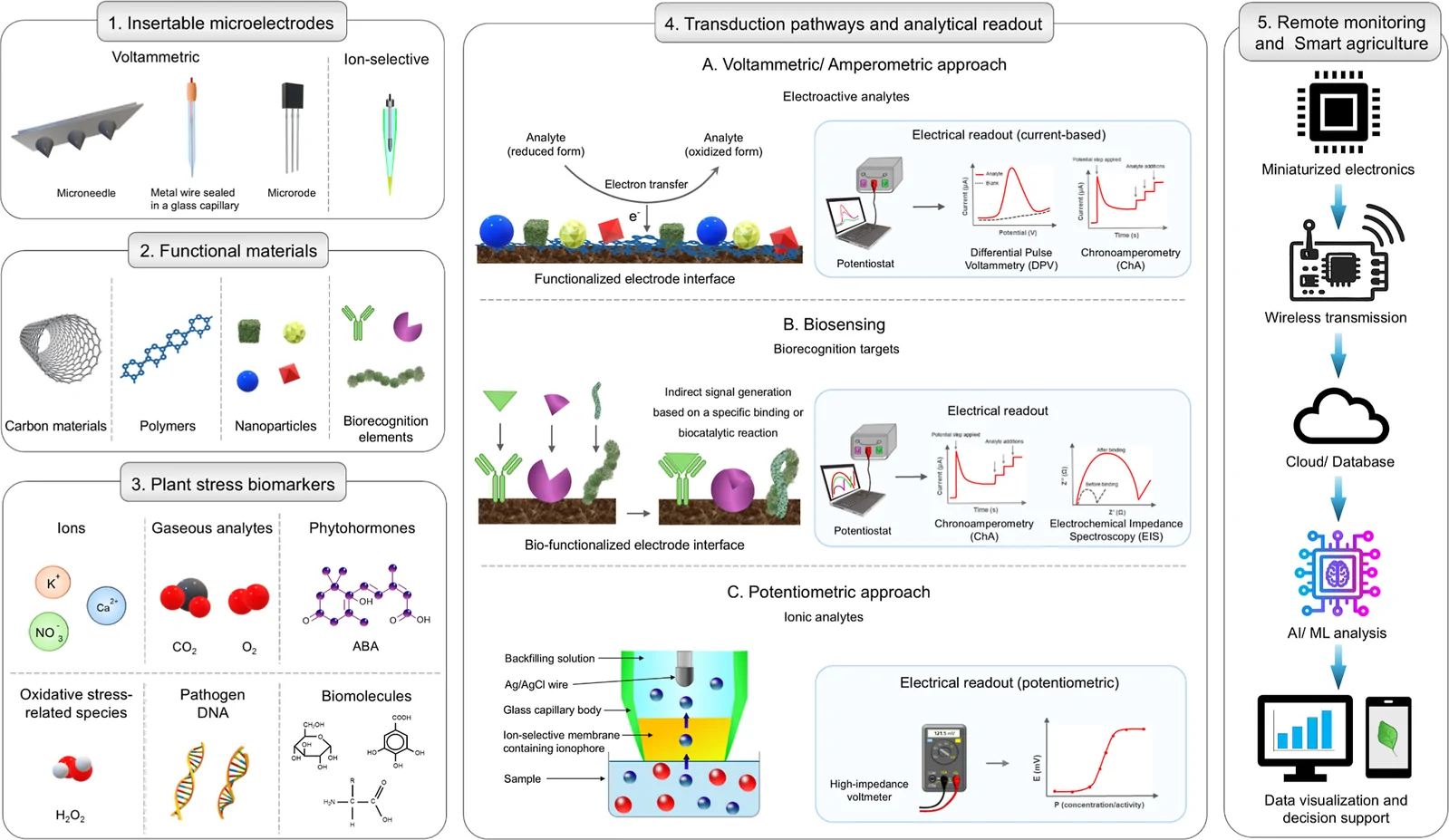
Schematic summary of insertable electrochemical microsensor components, sensing mechanisms, target biomarkers, and remote monitoring in smart agriculture.

**Table 1:** Representative Electrochemical Sensors and Microsensors for Plant Biomarker Detection^a^

target marker	electrode material/modification	plant species	plant tissue/sample	detection limit	calibration range	ref
nitrate	ion-selective membrane/ChemFET	corn	stalk	not reported	0.0071–71.4 mM (log–linear response)	^34^
nitrate	C–GO–VB_12_ carbon electrode	corn	stalk	0.0032 mM	0.0032–0.161 mM (CV); 0.0032–32.3 mM (EIS)	^35^
nitrate	MTDDA·NO_3_/PVC nitrate-selective microelectrode	Chara corallina	internodal cells	0.50 mM in the presence of 100 mM Cl^–^	0.10–100 mM	^36^
intracellular free Ca^2+^, predominantly vacuolar	filamented borosilicate micropipette/Calcium Ionophore 1 cocktail A	apple	cortical cells	not reported	0.001–1.0 mM (linear region, pCa 3–6)	^37^
extracellular/apoplastic free Ca^2+^	filamented borosilicate micropipette/Calcium Ionophore 1 cocktail A	apple	osmoticum-filled sampling well cut through the fruit epidermis	not reported	0.001–1.0 mM (linear region, pCa 3–6)	^37^
ammonium	nonactin/KTCPB/DBS/PVC NH_4_^+^-selective microelectrode	Chara corallina	internodal cells; cytosolic and vacuolar measurements	9.4 mM in the presence of 72 mM K^+^	not reported	^38^
K^+^	LIG/PEDOT/PSS/K^+^-selective membrane	lettuce, tomato, and aloe vera	petiole, stem, and leaf	0.20 μM	1–400 mM	^39^
K^+^	carbon-coated SS MN/f-MWCNTs/K^+^-selective membrane/PU	basil	stem	not reported	covers 50–120 mM expected physiological interval	^40^
K^+^	GelMA hydrogel MN/PEDOT/PSS/K^+^-selective membrane	rice	leaf	3.5 × 10^–3^ mM	0.10–100 mM	^41^
Na^+^	SS MN/G/PEDOT/PSS/Na^+^-selective membrane	tomato and rice	stem	1.94 × 10^–6^ M	1.0 × 10^–5^ to 1.0 × 10^–2^ M	^42^
Na^+^	carbon-coated SS MN/f-MWCNTs/Na^+^-selective membrane/PU	basil	stem	8.8 × 10^–6^ M	0.010–180 mM	^40^
pH	LIG/AuNPs/PANI	lettuce, tomato, and aloe vera	petiole, stem, and leaf	not applicable	pH 2–9	^39^
pH	3D-printed HMNA/paper sampling cell/PANI-SPE	Pilea peperomioides and Echeveria “raindrops”	leaf apoplast fluid	not applicable	pH 5–8	^43^
ABA	Au@SnO_2_–VG/Ta MN array	cucumber	fruit	0.002–0.005 μM, depending on pH	0.012–495.2 μM; lower limit of 0.012 or 0.024 μM depending on pH	^44^
ABA	Au@Pt/TDN-functionalized MXene-FET glass MN	tomato	stem and leaf	100 aM, lowest reported responsive concentration	nonlinear Hill-type response; saturation near 100 μM	^45^
SA	Au@Cu_2_O–G–PDA-modified Al IDME array	cucumber	leaf punctured before application of the planar sensor	0.00116 μM	0.01–1.04 and 1.04–100 μM	^46^
SA	self-supporting NDG microelectrode/Pt wire/Ti tube concentric needle sensor	cucumber	fruit and stem	0.14–0.46 μM, depending on pH	1–500 μM	^47^
SA	Pt nanoflowers/ERGO/Pt microelectrode	sunflower seedlings	stem; 10% stem-homogenate calibration used for the in vivo analysis	4.81 × 10^–8^ mM	1.0 × 10^–7^ to 1.0 × 10^–3^ mM (log–linear)	^48^
IAA	PST/Pt–ERGO/Au/anodized SS microelectrode	soybean seedlings	stem	2.45 × 10^–7^ mM	5.71 × 10^–7^ to 0.571 mM (log–log linear)	^49^
IAA flux	MWNT-modified Pt self-referencing microelectrode	maize	root apex; noninvasive surface-flux measurement 2–12 μm from the tissue	1.0 × 10^–4^ mM	1.0 × 10^–3^ to 2.0 × 10^–2^ mM	^50^
IAA exocytotic release	TiC@C/Pt-QANFAs microdisk sensor	oilseed rape	single protoplasts isolated from young developing leaves; sensor placed against the cell surface	1.0 × 10^–6^ mM	1.6 × 10^–5^ to 1.0 × 10^–3^ mM	^51^
IAA	N-CNTs/core–shell Au@Cu_2_O/carbon–fiber microelectrode	flowering cabbage	stem	6.17 × 10^–8^ – 3.30 × 10^–7^ mM, depending on pH	5.71 × 10^–7^ to 5.71 × 10^–2^ mM (piecewise linear)	^52^
IAA	Au-coated polymer MN/POPDMIP	tomato	leaf	0.44 μM	0.50–50 μM	^53^
glucose	MPt–Au–Nafion–GOx–PU sensing interface integrated into a polymer HMN	tomato and aloe vera	tomato stem and Aloe vera leaf, in situ	0.0333 mM	0.10–10 mM	^54^
glucose	NPPt/GO/GOx/Nafion microelectrode	tomato and cucumber	fruit homogenate/filtrate	0.0237 mM	0.10–4.0 and 4.0–20.0 mM	^55^
glucose	3D-printed HMNA/paper sampling cell/PB–GOx SPE	Pilea peperomioides and Echeveria “raindrops”	leaf apoplast fluid	not reported	50–1500 μM	^43^
l-Trp	PSA/MWCNTs-COOH/GrRE	tomato	fruit in vivo and juice in vitro	3.7 × 10^–5^ – 1.21 × 10^–4^ mM, depending on pH	0.010–0.030 and 0.030–0.110 mM	^56^
free Trp	PDA/rGO–MnO_2_/GCE	tomato	fruit in situ and tomato juice in vitro; sensor unsuitable for small tissues because of its size	2.2 × 10^–4^ – 3.9 × 10^–4^ mM, depending on pH	0.001–0.300 mM	^57^
GA, model compound	CNT/CNC–PANI–GOPS-modified SS-MN	orange and kiwi	orange juice; orange and kiwi fruits used for combined GA/CA-equivalent depth profiling	(1.7 ± 1.2) × 10^–3^ mM	5.88 × 10^–4^ to 0.513 mM	^58^
CA, model compound	CNT/CNC–PANI–GOPS-modified SS-MN	orange and kiwi	orange juice; orange and kiwi fruits used for combined GA/CA-equivalent depth profiling	(9.6 ± 5.6) × 10^–4^ mM	2.82 × 10^–4^ to 0.220 mM	^58^
H_2_O_2_	Au/SS wire microsensor	tomato	Leaf vein	3.97 μM	10–1000 μM	^59^
H_2_O_2_	dual-function Pt disc microelectrode with a silver-epoxy pseudoreference	Agave tequilana	upper epidermis of the leaf; inserted into a small cavity made with a needle	not reported	0.10–100 mM (CV calibration)	^60^
H_2_O_2_, oxidative burst induced by Sclerotinia sclerotiorum infection	Pt/Pt/POPD electrode	oilseed rape (Brassica napus L.)	leaf; working electrode inserted into the leaf nervation	10 μM in 1 mM NaCl calibration solution; 40 μM in centrifuged leaf homogenate	not reported	^61^
H_2_O_2_	LIG/PtNPs/Nafion	lettuce, tomato, and aloe vera	petiole, stem, and leaf	0.60 μM	10–400 μM	^39^
H_2_O_2_	Au-coated PU MN/HRP–Cs-rGO biohydrogel	tobacco and soybean	leaf	0.06 μM	0.10–4500 μM, log–linear	^62^
H_2_O_2_	3D-printed HMNA/paper sampling cell/PB-SPE	Pilea peperomioides and Echeveria “raindrops”	leaf apoplast fluid	not reported	10–100 μM	^43^
H_2_O_2_	PI/LIGS/PtNPs/Nafion microsensor	tomato	stem	0.35 μM	2–200 μM in buffer; 5–200 μM in heat-treated tomato sap	^63^
H_2_O_2_, oxidative burst induced by UV-A/UV–C exposure	POPD/Pt-MP/Pt electrode	(Brassica napus L.)	leafstalk	8 μM (leaf homogenate)	24.4–1950 μM (leaf homogenate)	^64^
leaf bioimpedance	Ti/Ni/Au-coated PDMS MNA	barley	leaf blade	not applicable	Not applicable	^65^
stem sap electrical conductivity	silicon MN with Ti/Au interdigitated electrodes	tomato	stem, measured in vivo	not reported	0.084–12.800 mS cm^–1^ (standard solutions; log-transformed calibration at 10 kHz)	^66^
leaf bioimpedance	aerosol-jet-printed AgNP MN arrays on inkjet-printed AgNP/PET tracks	mint, jade tree, and jasmine	leaf	not applicable	not applicable; relative impedance changes during dehydration and ion uptake	^67^
P. infestans DNA and TSWV RNA	PVA MN patch combined with a smartphone LAMP/*RT*-LAMP system	tomato	laboratory-inoculated and field-collected leaves	1 pg	1 pg to 1 ng; threshold time was linear with log target amount	^68^
plant DNA and P. infestans DNA	PVA MN patch; real-time PCR performed after extraction^1^	tomato, potato and pepper	leaves; pathogen DNA detection was demonstrated in infected tomato leaves	not reported	not reported	^69^

a A list of abbreviations is provided at the end of the paper.

### 2.1 Ions

When plants are under abiotic stress, such as drought or salinity, they regulate ion homeostasis through tightly coordinated signaling. Salt stress induces Na^+^ influx through nonselective cation channels (NSCCs), which elevates cytosolic Ca^2+^, which binds to SOS3 (CBL calcium sensor) and forms the SOS3-SOS2 complex by activating the SOS2 kinase. SOS2 phosphorylates the plasma membrane Na^+^/H^+^ antiporter SOS1 to expel Na^+^, and Na^+^ is also sequestered into vacuoles by NHX transporters.^70,71^ K^+^ homeostasis is maintained by HAK/KUP/KT transporters and channels such as AKT1. The activities of AKT1 and HAK5 are regulated by CBL–CIPK complexes, particularly CIPK23-containing complexes, while HAK/KUP/KT transporters contribute to K^+^/Na^+^ homeostasis, salt tolerance, and osmotic adjustment. During K^+^ deficiency, increased cytosolic Ca^2+^ acts as a regulatory signal associated with CIPK23-dependent activation of AKT1 and probably HAK5.^72^ Under water stress, nitrate reduction through nitrate reductase (NR) and nitrite reductase (NiR), as well as glutamine synthetase (GS)- and glutamate synthase (GOGAT)-mediated ammonium assimilation, can decline. Reduced GS/GOGAT activity may consequently contribute to ammonium accumulation and toxicity.^73^ Therefore, understanding the precise intracellular free ion concentrations is crucial for understanding cellular signaling, nutrition, metabolism, membrane transport, and the pathways through which plants respond to unfavorable environmental conditions.^74,75^ Ion-selective microelectrodes (ISMEs) have been widely used for the direct and continuous measurement of intracellular free ions in plant systems.^37,76^ The key component of an ISME is a lipophilic membrane with ion-selective properties. The membrane contains ionophores or ion-exchange sites that selectively interact with the target ion, generating a potential difference across the membrane. This potential is proportional to the logarithm of the activity of the target ion (Figure 2A).

**Figure 2:**
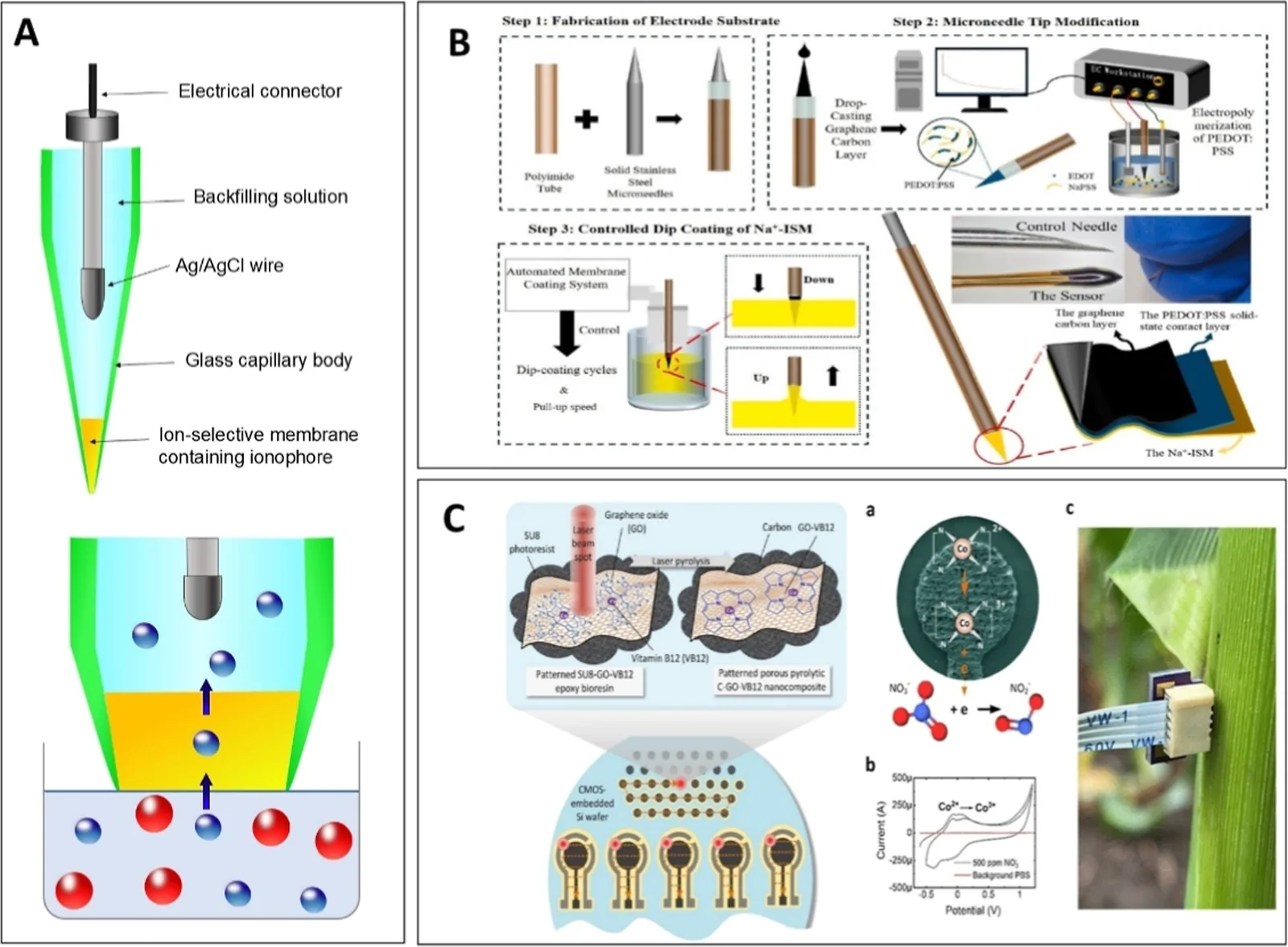
(A) Components of a liquid-contact ISME and selective ion transport through ionophore-containing membrane; (B) A solid-contact Na^+^-MN sensor prepared by coating the MN with a solid contact layer consisting of G and PEDOT/PSS, followed by coating with a Na^+^-selective membrane, Reproduced with permission from.^42^ Copyright 2025 Elsevier; (C) Fabrication of a carbon–GO–VB_12_ nanocomposite needle sensor for in planta nitrate detection in living maize, along with its functional mechanism based on the redox-mediating activity of the Co­(II)/Co­(III) couple, Reproduced from.^35^ Copyright 2022 American Chemical Society.

Among mineral nutrients, Ca^2+^ may be the most intriguing. As reported by Harker and Venis,^37^ intracellular and extracellular free Ca^2+^ were measured in cortical cells from apple fruit using Ca^2+^-selective microelectrodes. Since placing microelectrodes in the cytoplasm was challenging, most intracellular measurements reflected vacuolar Ca^2+^ concentrations. Extracellular free Ca^2+^ measurements were performed in wells cut through the fruit epidermis and then filled with osmoticum. To measure nitrate directly, Miller and Zhen,^36^ developed nitrate-selective microelectrodes utilizing the quaternary ammonium methyl-tridodecylammonium nitrate as the ion exchanger in a poly­(vinyl chloride) (PVC) matrix. The nitrate levels in *Chara corallina* internodal cells cultivated under low-nitrate and nitrate-replete conditions were also measured using these microelectrodes because of their broad linear range spanning from 0.1 mM to 0.1 M. Cells grown under low-nitrate conditions showed values below the detection limit of the electrode. Ammonium-selective microelectrodes also offer a method for extracellular measurements.^77^ The greatest issue with intracellular measurements is interference from K^+^. However, these electrodes were used to measure cytosolic and vacuolar NH_4_^+^ activities in *C. corallina* cells incubated in an ammonium-containing nutrient solution. The macrotetralide antibiotic nonactin served as the ammonium-selective ionophore in these electrodes.^38^ In another report, ISMEs were used in an experimental system to measure electrochemical potential gradients of H^+^ and K^+^ ions in the unstirred layer near the root surface of intact corn seedlings and corn root segments. Simultaneous measurement of fluxes across a specific area of the root surface was enabled by analyzing steady-state H^+^ and K^+^ electrochemical potential gradients. Compared with the unidirectional K^+^ (^86^Rb^+^) influx kinetic data, the net K^+^ influx values obtained by this method were comparable to those obtained at the same K^+^ concentration. The relationship between net H^+^ efflux and net K^+^ influx was then examined using an ISME device. The results suggested that a K^+^–H^+^ cotransport mechanism, rather than a K^+^/H^+^ antiporter, may transport K^+^ into corn roots.^78^ To study ion transport across maize seedling roots, Xue et al.^79^ created a K^+^-selective microelectrode utilizing liquid ion exchangers. In the calibration experiments, the K^+^ fluxes changed only slightly, suggesting that the ion flux measurement system is highly stable. Direct pH measurement in plant cells can also be a promising approach for early diagnosis and disease management. Therefore, Kurkdjian and Barbier–Brygoo,^80^ presented an H^+^-selective liquid–membrane microelectrode to detect proton concentrations in the vacuoles of plant cells. The microelectrodes retained their slope and electrical resistance for up to 48 h of storage, whereas the H^+^-selective liquid retained its sensitivity after 120 days of storage. Although ISMEs have enabled valuable intracellular and extracellular ion measurements, the presence of an internal electrolyte limits their mechanical robustness and long-term stability. In solid-contact ion-selective electrodes (SC-ISEs), the internal filling solution is replaced by a solid ion-to-electron transducer, such as a conducting polymer or carbon nanomaterial, positioned between the electronic conductor and the ion-selective membrane. This solid-contact layer converts the ionic signal generated at the membrane into an electronic signal while eliminating the need for an internal filling solution. Consequently, SC-ISEs exhibit a simpler structure, improved mechanical stability, greater potential for miniaturization, a lower risk of electrolyte leakage, and enhanced potential stability,^81,82^ making them more suitable for insertable sensing platforms. Fan et al.^42^ reported the development of a SC-ISE for the in vivo determination of Na^+^ in tomato stem and rice seedling. The sensor was fabricated using a stainless-steel microneedle (SS-MN) as the electronic conductor. A solid contact layer consisting of poly­(3,4-ethylenedioxythiophene)/poly­(styrenesulfonate) (PEDOT/PSS) was then deposited on the MN surface through electropolymerization to facilitate ion-to-electron transduction and improve potential stability. Finally, the MN was coated with a lipophilic sodium-selective membrane containing a Na^+^ ionophore to impart selective recognition of sodium ions (Figure 2B). The solid contact layer of PEDOT/PSS improved the sensitivity and stability of the sensor response by enhancing the charge transfer efficiency. An SC-ISE for detection of K^+^ in lettuce stem was developed by Zhai et al.^83^ through electrodeposition of a layer of MnO_2_ onto a SS-MN followed by coating with a K^+^ selective PVC-based membrane. The fabricated microsensor showed a short response time of 15 s, enabling real-time monitoring.

In addition to potentiometric ISMEs, an impedimetric microsensor has also been developed for the determination of nitrate ions in living maize plants. In a study reported by Ibrahim et al.,^35^ a photosensitive epoxy bioresin was employed to fabricate high-resolution conductive microelectrodes with vitamin B_12_ (VB_12_) and graphene oxide (GO) embedded directly within the sensing material (Figure 2C). The fabricated microsensor detects nitrate through the biomimetic catalytic activity of VB_12_. The Co­(II) centers in VB_12_ react with nitrate and are oxidized to the Co­(III) oxidation state, which is subsequently electrochemically reduced back to the catalytically active Co­(II) state. Therefore, increasing the nitrate concentration promotes the electrocatalytic redox cycle of the VB_12_ cobalt center, which subsequently facilitates interfacial electron transfer, leading to reduced charge-transfer resistance. The dimension and geometry of the prepared microsensor enable feasible insertion into the plant for in vivo measurement. Furthermore, the use of the enzyme mimetic VB_12_ instead of the natural enzyme NR results in higher sensor stability and reusability.^84,85^

The studies discussed in this section on the development of ISMEs showed that these microelectrodes are useful for investigating cytosolic ion activity in single cells and surface cells of plant tissues. ISMEs are potentiometric sensors that utilize selective membranes to measure the activity of specific ions in complex biological environments. However, one of the main limitations of intracellular measurements with these microelectrodes is that they provide information only from a specific point within the cell. This can lead to an incomplete picture of the cell’s true state when significant ion gradients exist in the cytoplasm. Furthermore, this method is usually limited to single cells accessible near the tissue surface, requires specialized equipment and skilled operator, and is too slow to detect rapid transient changes because of its relatively long response time (on the order of minutes). Despite these limitations, ISMEs have significant advantages. This method allows the measurement of ion activity in living cells with minimal disruption and without altering the activity of the target ion. Additionally, measurements with ISMEs are performed under equilibrium conditions, eliminating the need for the application of an external excitation potential. Furthermore, these sensors allow the simultaneous measurement of electrical and chemical gradients across biological membranes. Taken together, these features make ISMEs a valuable tool for studying ion homeostasis, membrane transport, and electrochemical processes in plant systems.^86,87^

### 2.2 Phytohormones

Plant hormones (phytohormones) are essential for regulating various biochemical and physiological processes that control plant development and productivity under both optimal and stressful conditions. As they activate signaling pathways, their coordinated action is critical for plant survival under environmental stress.^88^ The levels of numerous phytohormones are altered in response to external stimuli. Abscisic acid (ABA), gibberellins (GA), auxins, cytokinins (CK), salicylic acid (SA), ethylene (ET), brassinosteroids (BR), strigolactones, and jasmonates (JA) are the main hormones that plants produce. ABA, SA, JA, and ET play significant roles in modulating plant defense response to pathogens and abiotic stressors.^89^ As ABA levels increase in response to heat, cold, salinity, and drought stress, plant defenses against abiotic stresses are frequently mediated by the ABA signaling pathway.^90,91^ Therefore, ABA is now recognized as a plant stress hormone, and its in situ measurement is worthwhile. For this purpose, Au@SnO_2_-vertical graphene (VG)/Ta microelectrodes were fabricated by Wang et al.^44^ for in situ analysis of ABA in cucumbers. A Ti wire was used as a reference electrode, a Pt wire served as a counter electrode, and three Au@SnO_2_–VG/Ta microelectrodes were integrated into a three-electrode microneedle array (MNA) sensor. The ABA content was subsequently quantified by direct electrocatalytic oxidation, which takes advantage of the numerous catalytically active sites in the Au@SnO_2_ nanoparticles and the superior conductivity of the VG nanosheets. The small size, broad pH operating range (4–7), low detection limit (2 nM), and wide linear range (0.012 to 495.2 μM) make the developed microsensor well suited for in situ detection of ABA.

Crosstalk between ABA and JA signaling pathways is involved in regulating plant stress responses.^92^ Under drought stress, upregulation of NCED genes increases ABA biosynthesis and accumulation.^91,92^ ABA perception by PYR/PYL/RCAR receptors inhibits PP2Cs, thereby leading to the activation of SnRK2 protein kinases.^93,94^ SnRK2 regulates RBOH activity, which generates superoxide (O_2_^•–^) by transferring electrons to apoplastic oxygen. The produced O_2_^•–^ is subsequently converted to H_2_O_2_ by superoxide dismutase (SOD).^94^ Hydrogen peroxide (H_2_O_2_) is sensed by HPCA1, leading to HPCA1 autophosphorylation, Ca^2+^ channel activation, Ca^2+^ influx into guard cells, and promotion of stomatal closure.^95^ In the JA pathway, activation of JA signaling results in COI1-dependent degradation of JAZ repressors and the release and activation of MYC2.^96^ MYC2-dependent VSP2 expression contributes to anti-insect defense, while recombinant VSP2 has anti-insect activity and is associated with delayed insect development and increased insect mortality.^94,97^ At the same time, MYC2 reduces root meristem activity and primary root growth by directly repressing PLT1 and PLT2 expression, thereby demonstrating a link between defense responses and growth inhibition.^98^ A reported interaction between PYL6 and MYC2 provides a specific molecular link between these pathways (Figure 3).^92^ As an endogenous plant hormone, SA is important for plant maturation, growth, and stress and disease resistance.^99,100^ For example, SA content is significantly higher under various stresses and throughout the plant growth cycle. Increased activity of the enzymes phenylalanine ammonia-lyase and benzoic acid 2-hydroxylase has been found to cause SA accumulation in response to both water deprivation and ultraviolet B (UV–B) radiation.^101^ Therefore, monitoring SA levels in plants can serve as a basis for controlling plant growth and for better understanding their growth and health.^47^ Liu et al.^46^ generated an interdigitated microelectrode (IDME) array electrochemical sensor using a three-electrode system to measure SA in cucumber leaves (Figure 4A). Core–shell Au@Cu_2_O-graphene­(G)-polydopamine (PDA) nanoparticles were deposited onto Al microelectrodes by electrodeposition. Owing to the synergistic electrocatalytic activity of PDA, Au@Cu_2_O, and G and the high-density configuration of the three-electrode unit with microelectrodes, the sensor exhibited a sensitive differential pulse voltammetry (DPV) response based on the electrocatalytic oxidation of SA. Zhang et al.^47^ developed a concentric three-electrode needle sensor based on a self-supporting nitrogen-doped graphene (NDG) microelectrode and assembled four sensors into a parallel array for in situ SA detection (Figure 4B). SA was quantified by DPV using the first-scan oxidation peak at approximately +0.88 V versus Ti, and the four-sensor array exhibited approximately twice the sensitivity of a single needle sensor. Additionally, for in vivo detection of SA in salt-stressed sunflower seedlings, Pt wire microelectrodes modified with Pt nanoflowers and electrochemically reduced graphene oxide (ERGO) were used. This microsensor was subsequently used to detect SA in sunflower seedling stems under various salt stressor conditions in vivo. The results demonstrated that SA levels decreased as the salt concentration increased.^48^ Practical application requires the integration of the working and reference electrodes into a single unit, as well as further miniaturization. Auxin, mainly represented by indole-3-acetic acid (IAA), is a widely distributed phytohormone that affects practically every aspect of plant growth and development, including how crop plants respond to drought and salt stress. Abiotic stresses, such as drought and salinity, affect auxin transport and distribution. Stress conditions can alter the expression of auxin transporters, particularly PIN-formed proteins, which play crucial roles in modulating root architecture and growth by influencing auxin distribution. In this context, a platinum (Pt) microelectrode modified with nanomaterials was developed to detect IAA isolated from whole plant tissues. A layer of Pt black, overnight silanization, and a coating of multiwalled carbon nanotubes (MWCNTs) were used to modify the nanoscale electrochemical interface. Using a three-electrode setup, an electroanalytical assessment of sensor performance was carried out with both IAA standards and IAA isolated from maize plant samples. Additionally, tissue concentrations were evaluated in maize seedlings exposed to salt and water stress, and the results were compared with those obtained using standard ELISA techniques. The electrochemical biosensor was found to be more sensitive and reliable. Although the sensor showed promising analytical performance in both buffer solutions and plant tissue extracts, the use of a conventional three-electrode setup with macroscopic electrodes limits its applicability for in vivo analysis. A disposable stainless steel (SS)-based electrochemical microsensor was prepared by Li et al.^49^ to detect IAA under salt stress in soybean seedling stems. The results reveal that IAA signaling plays an important role in soybean responses to salt stress. Subsequent modification with Au nanostructures, platinum nanoparticles (PtNPs), ERGO, and poly­(safranine T) (PST) film (Figure 4C) resulted in a low detection limit of 43 pg mL^–1^ for the developed microsensor. A MWCNT-modified Pt microelectrode for noninvasive and continuous monitoring of auxin fluxes in the intact root apex was demonstrated by Mancuso et al.^50^ Chronoamperometric determination of IAA was achieved by applying a potential of +0.7 V vs Ag/AgCl, corresponding to the diffusion-limited oxidation of IAA, and monitoring the resulting oxidation current. Indeed, the electrocatalytic activity of the MWCNTs facilitated oxidation of IAA. Another electrochemical microsensor, PtNPs-modified titanium carbide–carbon core–shell structure/quasi-aligned nanofiber arrays (TiC@C/QANFAs), was fabricated for real-time monitoring of IAA efflux by vesicular exocytosis from single plant protoplasts (Figure 4D). This highly sensitive microsensor was fabricated by uniformly dispersing high-density, small PtNPs on TiC@C nanofiber arrays.^51^ The study did not investigate critical analytical parameters required for practical plant sensing, including lifetime, electrode fouling, and long-term stability. In general, electrochemical microsensors for phytohormone detection are based on amperometric or voltammetric techniques and often employ nanostructured materials with high catalytic activity to enhance sensitivity. These sensors can be used for in vivo and in situ monitoring of phytohormones with high sensitivity and relatively fast response times, making them well-suited for studying dynamic hormonal regulation under stress conditions. However, their performance is often limited by insufficient selectivity, resulting from structural similarities among phytohormones, interference from coexisting biomolecules in the plant matrix, and fouling of the electrode surface. Compared to conventional analytical methods, microsensors offer significant advantages, including real-time monitoring without sample preparation, though achieving high selectivity requires careful functionalization of the electrode surface. These features make microsensors suitable tools for monitoring hormonal signaling in plants under stress conditions. An important limitation of voltammetric and amperometric sensors is the requirement to apply an external potential to drive the analyte redox reaction. The required potential may be sufficient to induce undesired electrochemical reactions or perturb plant physiology, affecting tissue viability during prolonged in vivo measurements.

**Figure 3:**
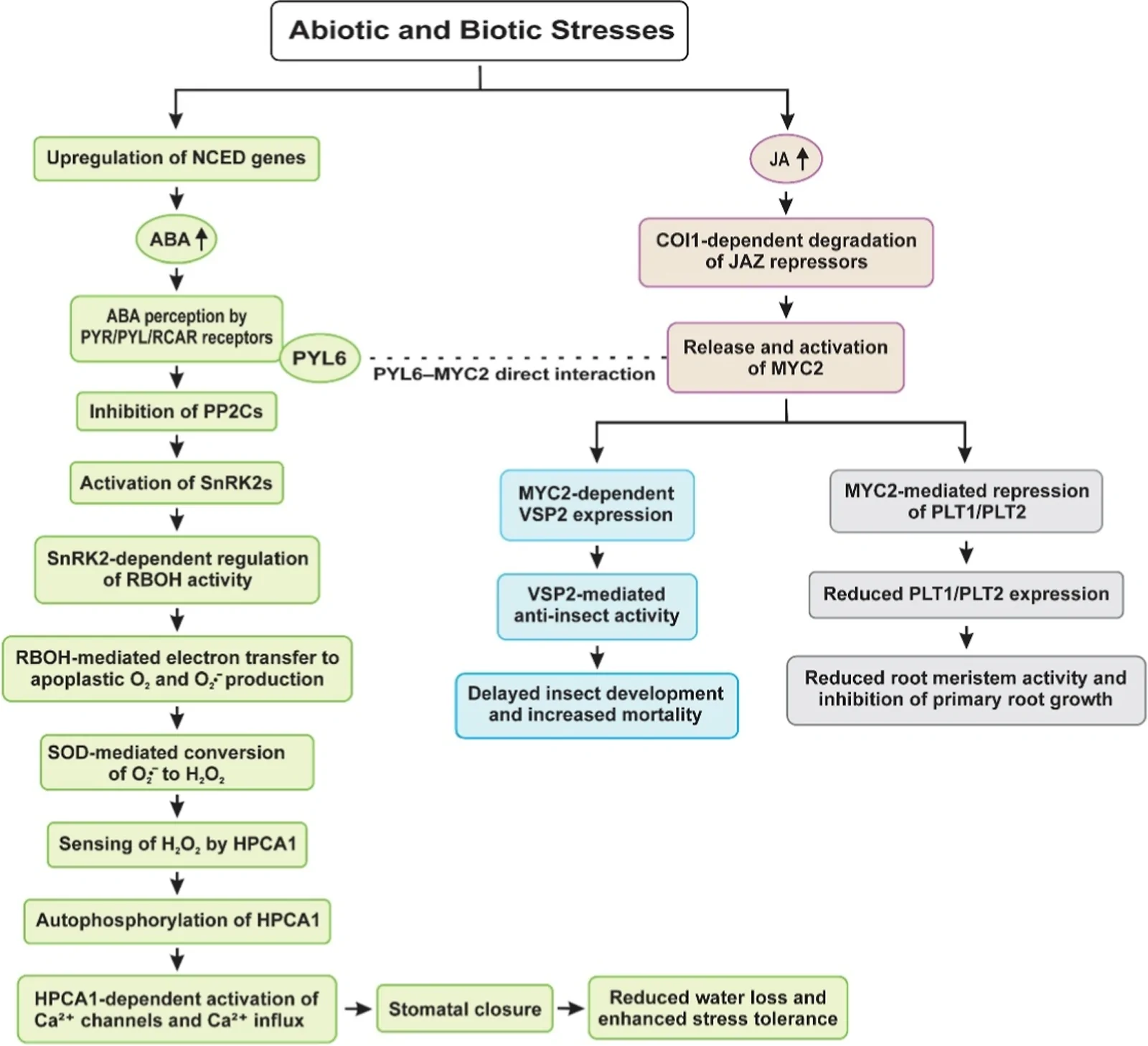
Schematic representation of ABA and JA signaling pathways in responses to plant stresses. The ABA pathway is associated with the induction of ROS production, Ca^2+^ influx, and stomatal closure, while the JA pathway regulates MYC2-dependent defense against insects and growth inhibition. The dashed line indicates the crosstalk between the two pathways through the reported PYL6–MYC2 interaction.

**Figure 4:**
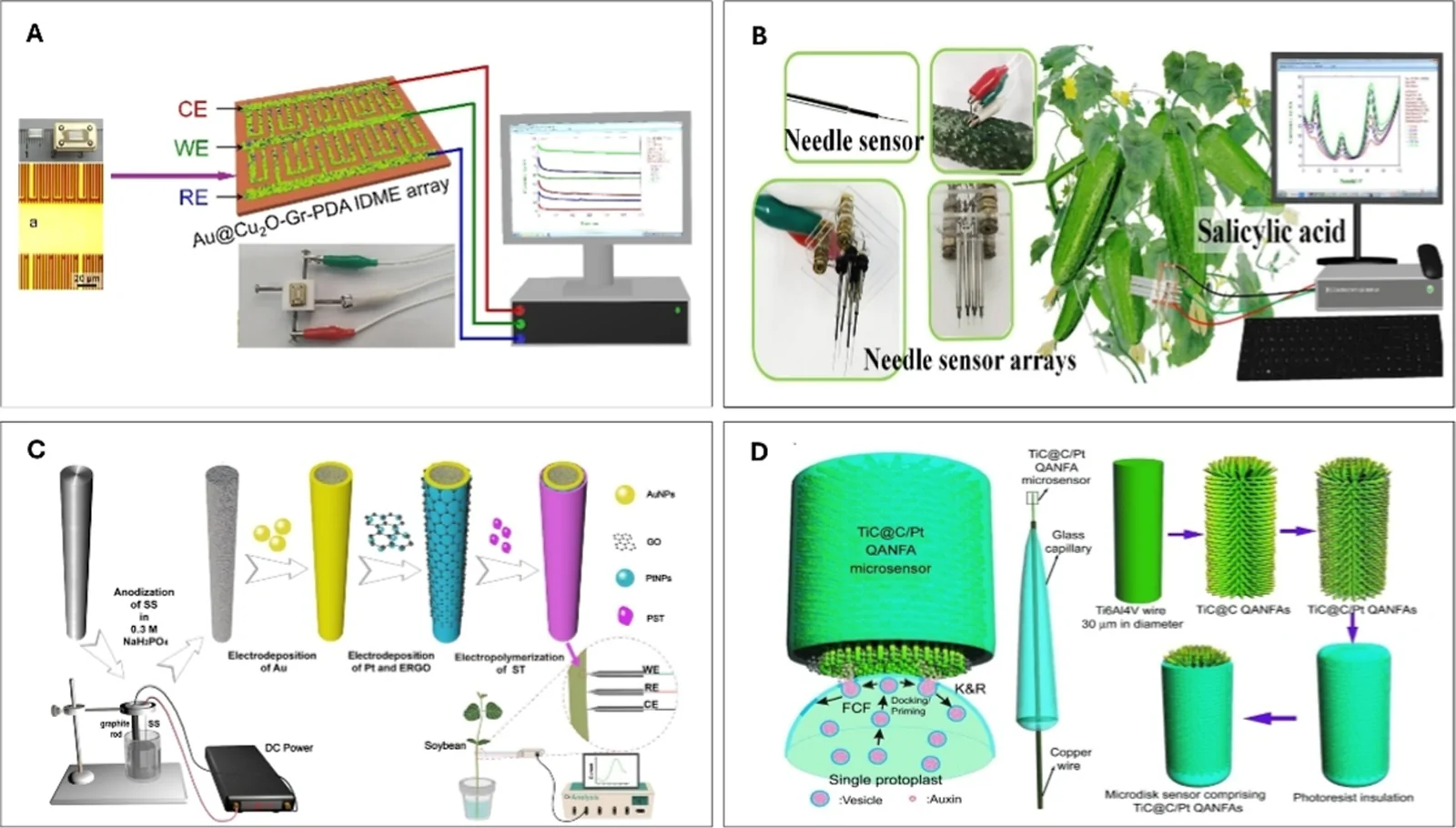
(A) An IDME array including Au@Cu_2_O–G–PDA modified working electrode configured for in situ determination of SA in cucumber leaves, Reproduced with permission from.^46^ Copyright 2021 Elsevier; (B) A sensor array of four NDG modified MN for SA analysis in cucumber, Reproduced with permission from.^47^ Copyright 2021 Elsevier; (C) Sequential steps in the preparation of a disposable SS microelectrode by anodization, electrodeposition of AuNPs, coelectrodeposition of PtNPs and ERGO, and electropolymerization of ST for in vivo determination of IAA in soybean seedlings, Reproduced with permission from.^49^ Copyright 2018 Elsevier; (D) Sequential modification of a Ti6Al4 V wire and its subsequent insertion into a glass capillary to prepare a microsensor for amperometric monitoring of vesicular IAA efflux from a single plant protoplast, Reproduced with permission from.^51^ Copyright 2014 Wiley-VCH Verlag GmbH & Co. KGaA, Weinheim.

### 2.3 Gaseous Analytes

The study of gas exchange during photosynthesis in vivo has become an essential tool for analyzing plant responses to environmental changes, including pollution, drought, elevated atmospheric carbon dioxide (CO_2_) concentrations, temperature stress, and biotic stressors.^101^ Rapid and accurate quantification of physiological oxygen (O_2_) distribution is crucial for understanding the dynamics of temporally and spatially separated metabolism and specialized functions linked to stress signaling in cells and tissues.

Molecular O_2_ concentrations can be detected using amperometric or optical microsensors. The majority of O_2_ microsensors are scaled-down Clark-type sensors.^102,103^ Although Clark-type O_2_ microsensors consume only a negligible amount of O_2_, this consumption makes them slightly sensitive to stirring.^102,104^ In contrast, O_2_ micro-optodes neither consume O_2_ nor exhibit stirring sensitivity. However, the flexible nature of their glass-fiber probes makes direct insertion into plant tissues difficult. This limitation is less relevant to Clark-type O_2_ microsensors, because their tips can be tiny, and the rigidity of the glass capillaries enhances their suitability for insertion into plant tissues.^105^ The Clark-type O_2_ microsensor consists of a Pt working cathode, positioned behind an O_2_-permeable silicone membrane, together with an internal Ag/AgCl reference electrode immersed in 1 M KCl electrolyte. Upon application of a polarization potential (typically –0.8 V vs Ag/AgCl), O_2_ diffuses through the membrane and is electrochemically reduced at the Pt cathode. The resulting reduction current is proportional to the O_2_ partial pressure (or concentration) in the surrounding medium, enabling quantitative O_2_ measurements (Figure 5A).^104^

**Figure 5:**
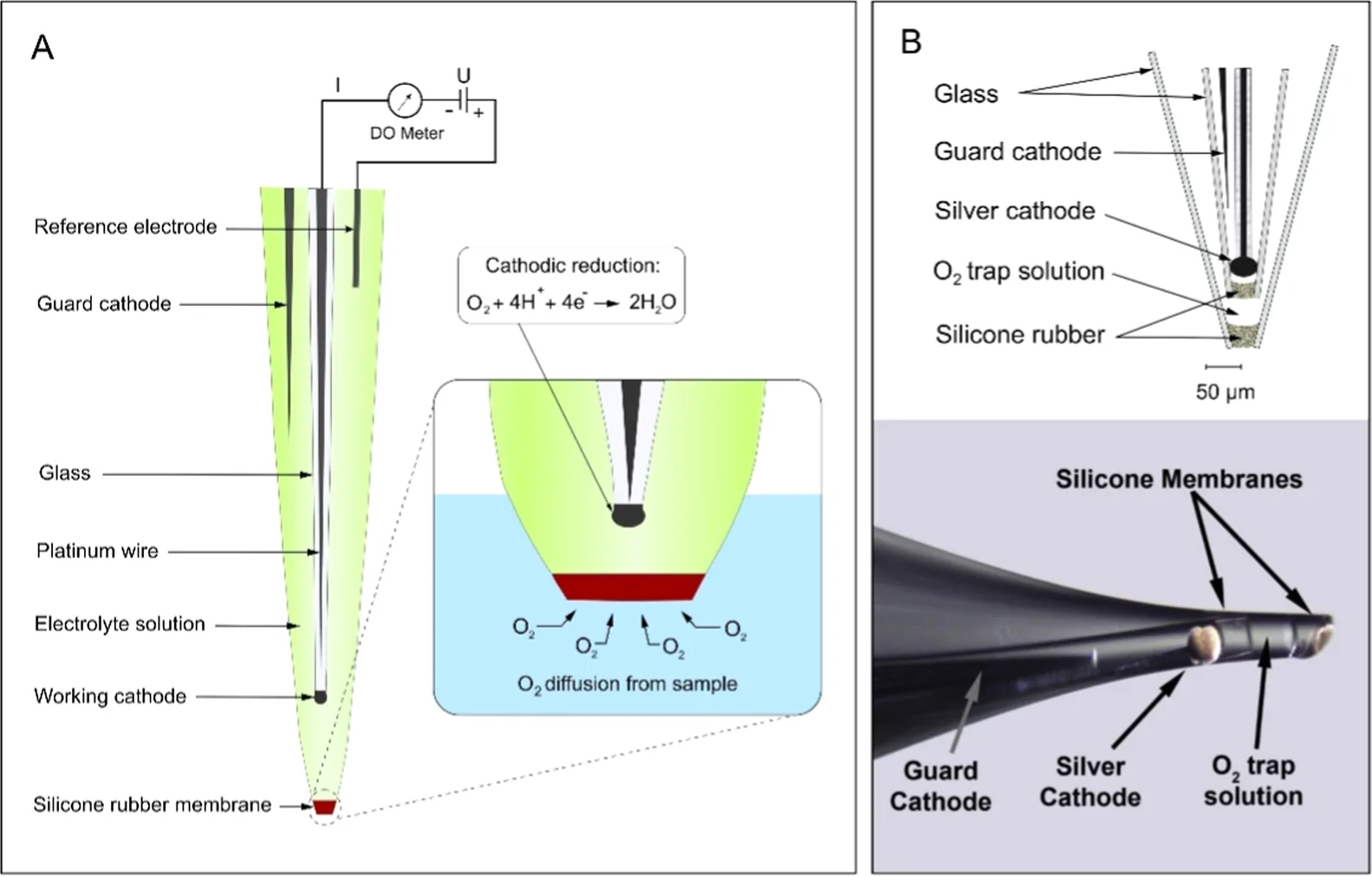
(A) Components of Clark-type amperometric O_2_ microsensor: O_2_ diffuses through the O_2_-permeable membrane and is reduced at the cathode, generating a concentration-dependent current; (B) Components of an amperometric CO_2_ microsensor, showing the front O_2_-trap compartment that prevents O_2_ from reaching the CO_2_-reducing cathode, Reproduced/adapted from ref 113 Available under a CC BY 4.0 license. Copyright 2019 Revsbech et al.

An exciting advancement in the quest to further miniaturize O_2_ sensors is the use of O_2_ nanoelectrodes based on carbon-filled quartz micropipettes with Pt-coated tips.^106^ The carbon nanoelectrodes had diameters of 100–500 nm, compared with the 3 μm tip diameter of a miniaturized Clark-type O_2_ microsensor.^106,107^ To date, their use has been limited to time-series measurements with a temporal resolution of 6 s^106^ In comparison, Clark-type O_2_ microelectrodes with a 90% response time of 0.3–0.4 s have been reported.^108^ A custom-made Clark-type O_2_ microsensor was employed to quantify O_2_ levels in the hypoxic niche of the *Arabidopsis* shoot apical meristem. The microsensor had a bevelled tip with a diameter of 3 μm and was used to obtain in vivo O_2_ profiles through the shoot apex of four- and seven-day-old seedlings. The measurements revealed a hypoxic region in the shoot apex that broadly overlapped with the stem-cell niche. Low O_2_ levels in the hypoxic niche regulate the rate of new leaf production by increasing the stability of ZPR2, a protein involved in organ polarity and shoot apical meristem activity. To maintain normal meristem activity, the stem-cell niche requires hypoxic conditions that regulate leaf production.^107^

A Clark-type O_2_ microsensor was also used to produce high-resolution tissue O_2_ profiles in chickpea roots, revealing the formation of an anoxic core as the external O_2_ supply decreased.^109^ A combination of low tissue porosity, which results in low diffusivity, and a higher O_2_ demand within the vascular cylinder than in the surrounding porous cortex leads to a significant decline in tissue O_2_ status within the vascular cylinder. Radial O_2_ profiles in plant roots have been investigated in only a few studies to date, and these have focused on monocots.^110^

High CO_2_ concentrations in roots can adversely affect cellular pH. However, little is known about CO_2_ concentration in roots, which is a significant knowledge gap.^109^ Therefore, the development of rapid and simple methods to address this need is very important. As a result, Severinghaus-type CO_2_ microsensors have been developed.^111,112^ However, they rely on indirect CO_2_ measurements made possible by changes in pH, which are detected by a pH-sensitive transducer enclosed in a small reservoir containing bicarbonate and carbonic anhydrase to accelerate CO_2_ hydration. Nevertheless, these sensors respond slowly to low CO_2_ concentrations and inaccurately to high CO_2_ concentrations, particularly in a logarithmic form.^112^ Hence, there are many restrictions on how these sensors can be used. To address this issue, an amperometric sensor for CO_2_ detection was developed by placing a layer of an acidic O_2_-trap solution containing Cr^2+^ in front of a Clark-type electrochemical sensor to chemically remove O_2_, allowing CO_2_ to be selectively electrochemically reduced in an ionic liquid medium. In addition, the acidic Cr^2+^ solution removes residual O_2_, while the guard cathode reduces interference from reducible contaminants within the sensor (Figure 5B).^113^ The prepared CO_2_ sensor exhibited a broad linear range spanning from 20 Pa to 6 kPa and high stability, enabling continuous measurements for up to four months. At CO_2_ concentrations below or above the working range, the sensor sensitivity declined.

Infrared gas analyzers (IRGAs) are frequently used to detect CO_2_ exchange between plant tissues and the environment, and stomatal conductance can be used to estimate intercellular CO_2_ concentrations. In contrast, the CO_2_ microsensor allows direct measurement of the tissue CO_2_ concentration.^114^ A high temporal resolution analysis of CO_2_ and O_2_ dynamics in the leaf tissues of *Lobelia dortmanna* and *Littorella uniflora* plants was performed using a novel CO_2_ microsensor in conjunction with a conventional O_2_ microsensor. According to previous studies, *p*CO_2_ builds up inside the leaves of *L. dortmanna* throughout the night and eventually reaches 3.5 kPa. Fixation into malate keeps CO_2_ in the *L. uniflora* plant at or below the sensor’s detection limit for most of the dark period. After that, CO_2_ also increased in *L. uniflora* plants. Nighttime leaf *p*O_2_ levels in both *L. dortmanna* and the *L. uniflora* were below air equilibrium, with *L. dortmanna* becoming severely hypoxic. During the day, malate decarboxylation led to higher CO_2_ levels in *L. uniflora* but lower CO_2_ levels in *L. dortmanna*. Interestingly, leaf pO_2_ in *L. uniflora* was high during the early light period but declined as the malate-derived CO_2_ supply was depleted. The study’s exceptional temporal resolution provided insights into the differences in photosynthesis between *L. dortmanna* and *L. uniflora*, as reflected in tissue CO_2_ and O_2_ dynamics.^115^

The CO_2_ sensor was recently used to measure respiratory CO_2_ fluxes between roots and the surrounding media and to provide the first radial concentration profiles of CO_2_ in roots.^109^ CO_2_ efflux can be calculated from the CO_2_ concentration gradient in the diffusive boundary layer surrounding the root using a modified form of Fick’s first law.^109,116^ Research has shown that when the external O_2_ supply is reduced, the respiratory quotient of the roots increases.^109^ A thick layer of epiphytes on seagrass leaves dramatically depletes CO_2_ because the leaves and microalgae compete for CO_2_ during light exposure, according to research using a novel CO_2_ microsensor. As a result of epiphytic microalgae extending the diffusive boundary layer and increasing the diffusive resistance to CO_2_ exchange with the surrounding seawater, CO_2_ accumulated near the leaf surface throughout the night.^117^ In addition to CO_2_, waterlogged soils may contain phytotoxins such as H_2_S. The chemical equilibrium of sulfides is mainly controlled by pH, with ionic forms (HS^–^ and S^2–^) predominating at neutral and high pH and gaseous H_2_S predominating at low pH. Although both H_2_S- and S^2–^-specific microsensors are available, H_2_S has received increasing attention in plant biology because its diffusion and spread through porous tissues and aerenchyma are similar to those of O_2_ and CO_2_.^118,119^ ISMEs have been used for the ex vivo determination of endogenous H_2_S in plant extracts, while amperometric H_2_S microsensors have also been inserted into intact seagrass tissues for continuous in vivo monitoring.^118,120^

In general, the microsensors discussed in this section use different detection principles depending on the type of target gaseous analyte. O_2_ microsensors are primarily amperometric, and Clark-type sensors remain a widely used standard due to their high sensitivity, rapid response, and compatibility with tissue measurements. In contrast, Conventional Severinghaus-type CO_2_ microsensors rely on indirect pH-based detection, which can lead to slower response times and reduced accuracy, especially at high concentrations. Emerging nanoelectrode-based O_2_ sensors offer improved spatial resolution due to their smaller tip size. However, their practical application remains limited due to lower temporal resolution and technical challenges.

Compared with conventional approaches, microsensors enable direct, localized measurements in plant tissues, providing unique insights into gas dynamics at high spatial resolution. However, the performance of these sensors can be affected by factors such as O_2_ consumption during measurement, sensitivity to flow or stirring conditions, diffusion limitations, and mechanical challenges associated with inserting the sensor into plant tissues. Despite these limitations, their ability to provide direct, localized, and spatially resolved measurements makes them invaluable tools for investigating O_2_ and CO_2_ dynamics, as well as stress-related metabolic processes, in plants.

### 2.4 Biomolecules

Biomolecules include lipids, amino acids, carbohydrates, proteins, and nucleic acids, which are naturally found in living cells and contribute to their basic functions and structures.^121^ Amino acids are necessary for protein production and act as precursors for secondary metabolites involved in cell signaling, hormone synthesis, and protein phosphorylation. Conditions such as drought stress and salinity promote the accumulation of primary metabolites, including osmolytes, amino acids, and reducing sugars.^122^ Therefore, their quantification can help detect stress in plants at an early stage. Graphite rod electrode (GrRE) modified with MWCNTs and poly­(sulfosalicylic acid) (PSA) films were used by Yang et al.^56^ to fabricate an insertable electrode for in vivo detection of l-tryptophan (l-Trp) in tomatoes. More importantly, monitoring l-Trp levels across various growth phases may be beneficial for understanding the tomato growth process (Figure 6A). Such modifications lowered the oxidation potential of l-Trp, thereby reducing interference from electroactive species present in the plant matrix. However, validation using an independent analytical technique would further ensure the accuracy and reliability of the developed electrode. Gao et al.^57^ presented an insertable electrode by electrochemically depositing a PDA/reduced graphene oxide (rGO)-MnO_2_ nanocomposite onto a glassy carbon electrode (GCE; 10 × 20 × 1 mm) for in vivo detection of Trp. Owing to the combination of the selectivity of PDA and the catalytic activity of rGO-MnO_2_, the PDA/rGO-MnO_2_/GCE exhibited strong electrocatalytic activity for the selective oxidation of Trp (Figure 6B). A reliable Trp detection strategy was proposed that involved developing an appropriate quantitative detection model for the pH of the plant or plant tissue of interest, thereby addressing pH variability in plants. Additionally, the practical applicability of the PDA/rGO-MnO_2_/GCE-based sensor was successfully demonstrated by detecting Trp in tomato fruit and juice. The long-term stability of the sensor was evaluated over a one-month period, during which satisfactory analytical performance was maintained. Another study examined the possibility of an amino acid–H^+^ cotransporter in the coleoptiles of *Avena sativa L. cv*. “Garry” using microelectrode and tracer techniques. α-aminoisobutyric acid (AIB), an analog of amino acids, temporarily depolarizes the membrane potential. The same variables that affect AIB transport also affect the absolute magnitude of maximal depolarization. Both increased with increasing AIB concentrations and acidity and were enhanced by indole acetic acid (which hyperpolarized the membrane potential). AIB transport decreased as the K^+^ concentration in the medium increased and in the presence of the metabolic inhibitor NaN_3_, both of which reduced the membrane potential. These results are consistent with the amino acid–H^+^ cotransporter paradigm, in which the proton chemical potential difference across the coleoptile cell membrane, including proton concentration and membrane potential components, controls transport.^123^

**Figure 6:**
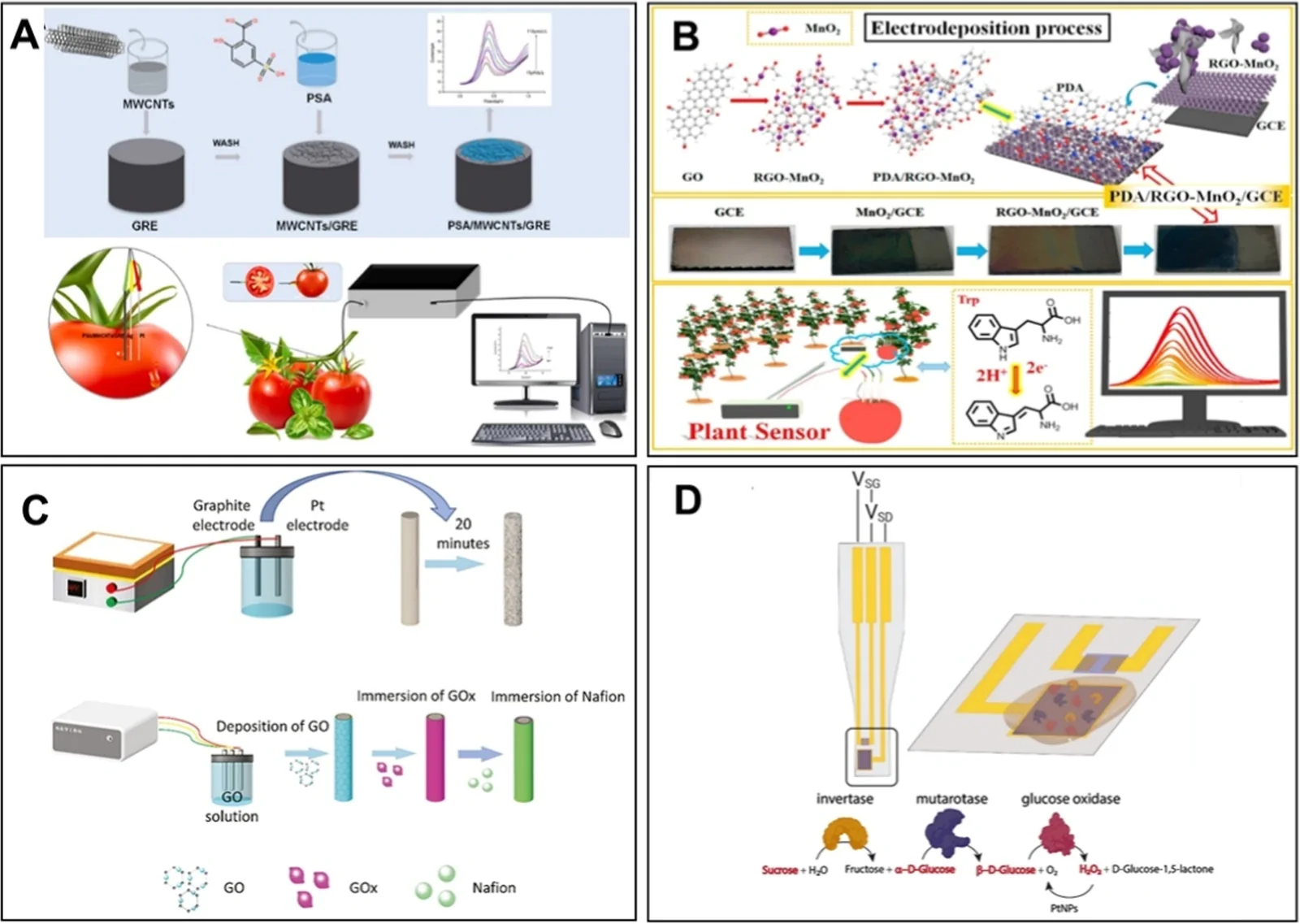
(A) Preparation procedure for modification of a GrRE with MWCNT and PSA to be used for in vivo detection of l-Trp in tomato fruit, Cropped and reproduced from ref 56 Available under a CC BY-NC-ND 4.0 license. Copyright 2021 Yang et al.; (B) Sequential modification of a GCE with MnO_2_, rGO, and PDA, for determination of Trp in tomatoes, Reproduced with permission from.^57^ Copyright 2020 Elsevier; (C) A glucose microbiosensor prepared by anodization of a Pt wire, subsequent electrodeposition of GO, and immersion in GOx and Nafion solutions, used for rapid glucose detection in cucumber and tomato fruits, Reproduced from ref 55 Available under a CC BY 4.0 license. Copyright 2022 Wu et al.; (D) In vivo sugar monitoring in trees using an OECT-based enzyme-cascade microbiosensor: oxidation of H_2_O_2_ at the gate alters the effective V_SG_, thereby modulating the source-drain current measured at an applied V_SD_, Reproduced from ref 129 Available under a CC BY-NC-ND 4.0 license. Copyright 2020 Diacci et al.

Throughout the entire growth cycle of photosynthetic plants, processes of carbohydrate synthesis, transport, metabolism, and storage are ongoing. These mechanisms are crucial for providing energy and signal transduction during plant growth and development. Plant development is controlled through the integration of internal regulatory variables, external environmental stimuli, and complex sugar metabolism and signal transduction networks. Glucose, produced by plant photosynthesis, is an aldohexose with five isomeric forms that serve as energy sources for different organs. Furthermore, as a critical carbohydrate regulatory signal, glucose plays crucial roles in signal transduction, primary and secondary metabolism, the cell cycle, and protein expression and regulation.^124^ Sugar levels in plant cells and their utilization, transport, and storage are tightly controlled and strongly influenced by cellular and organ physiological activity, environmental factors, circadian rhythms, and plant developmental phases.^125^ Invertases, hexokinases (HXKs), conserved glucose sensors, and variations in the adenosine monophosphate (AMP)-to-adenosine triphosphate (ATP) ratio may all play a role in the perception of signals produced by sugars in the apoplast, during transport across membranes, or inside the cell, such as in the cytosol.^126–128^ Therefore, a rapid and accurate method for detecting glucose in plants is urgently needed to properly assess plant stress responses, growth, and development. To rapidly detect glucose in tomato and cucumber fruits, Wu et al.^55^ prepared a microelectrode based on a nanoporous Pt/GO nanostructure (Figure 6C). For this purpose, a two-step modification was performed, including basic electrochemical etching of the Pt electrode surface and electrochemical deposition of GO. The resulting nanoporous Pt/GO structure increased glucose oxidase (GOx) loading, while Nafion helped encapsulate the enzyme on the electrode. Owing to its wide linear range of 0.1–20.0 mM, the biosensor accurately detected glucose. However, it should be noted that the incorporation of biological components, such as GOx, limits the lifetime of the electrode. Additionally, the enzymatic activity may vary from batch to batch, which can affect sensor reproducibility.

To monitor sugar fluctuations in tree vascular tissue in vivo and in real time, an enzyme-based organic electrochemical transistor (OECT) biosensor was developed (Figure 6D). OECT biosensors were inserted into the tree vascular tissue and operated for 48 h using an inexpensive portable unit to detect glucose and sucrose. In this proof-of-concept study, implantable OECT biosensors provided new insights into diurnal sugar homeostasis and enabled real-time monitoring of plant metabolites.^129^ Unlike conventional amperometric enzyme biosensors, which directly measure the current generated by redox reaction of the product of enzymatic reaction, the OECT converts the enzymatic reaction occurring at the gate electrode into a modulated transistor current, leading to signal amplification and enhanced sensitivity for continuous in vivo monitoring. Evaluation of the plant wound response following sensor implantation may be beneficial for future studies aimed at long-term monitoring.

Detecting biomolecules in plant systems requires a broader range of sensing approaches due to their structural and functional diversity. Among them, amino acids are often detected using amperometric or voltammetric methods, and nanostructured materials are utilized to enhance electrocatalytic activity. In contrast, carbohydrates, such as glucose, are mainly measured using enzymatic systems.

However, the chemical heterogeneity of biomolecules and the simultaneous presence of multiple metabolites in plant tissues pose serious challenges to sensor stability, especially in enzyme-based systems that are sensitive to environmental conditions. At the same time, the ability of these microsensors to provide in vivo and real-time data has made them key tools for investigating metabolic processes, material transport, and stress-related biochemical responses in plants.

### 2.5 Oxidative Stress-Related Species

Specific ROS signaling pathways, kinase cascades, and ion channels are triggered to reduce damage following stress exposure. Plants can adapt to and survive these stressful conditions because ROS accumulation promotes stress tolerance.^130^ ROS are continuously produced during multiple metabolic procedures in peroxisomes, chloroplasts, mitochondria, and the cytoplasm, and their excessive production disrupts normal metabolism.^131^ Redox reactions of molecules containing oxygen or nitrogen form reactive oxygen and nitrogen species (ROS and RNS). H_2_O_2_, hydroxyl radical (HO^•^), hypochlorous acid/hypochlorite (HOCl/OCl^–^), peroxyl radical (ROO^•^), O_2_^•–^, and singlet oxygen (^1^O_2_) are examples of ROS.^132,133^ Peroxynitrite (ONOO^–^), nitric oxide (^•^NO), and nitrogen dioxide (^•^NO_2_) are examples of reactive nitrogen species (RNS). Typical cellular processes, such as fatty acid catabolism, oxidative phosphorylation, breakdown of macromolecular compounds, phagocytosis, protein folding, and the generation of ROS and RNS, occur continuously. Each reactive species, which can be categorized as a radical (e.g., O_2_^•–^, NO^•^, or OH^•^) or nonradical (e.g., HOCl or H_2_O_2_), has distinct biological activity, reactivity rates, and functions inside the cell.^134,135^ Both radical and nonradical ROS contribute to the overall oxidative load of a cell.^133,135^ When present in excess, ROS and RNS can damage cellular components and induce oxidative stress due to their highly reactive nature.^136^ While the importance of ROS is generally accepted, the extent of their contribution to distinguishing healthy from pathological processes remains debated. Despite the importance of ROS/RNS, there are currently no appropriate analytical techniques for selectively monitoring changes in ROS/RNS levels to assess the cell’s oxidative status in situ.^137^ Electrochemistry offers exceptional opportunities to detect reactive species in living tissues using specially designed microelectrodes, enabling direct, real-time monitoring of ROS levels with excellent spatial resolution.^138–140^ H_2_O_2_, a critical signaling molecule, is crucial for numerous physiological processes in plants. H_2_O_2_ is generated through electron transport in mitochondria and chloroplasts, the dismutation of O_2_^•–^ by SOD, nicotinamide adenine dinucleotide phosphate (NADPH) oxidases in the plasma membrane, other apoplastic oxidases, peroxisomal oxidases, and type III peroxidases.^131^ The in vivo modulation of H_2_O_2_ could be continuously monitored to better understand this process. A disposable electrochemical microsensor for H_2_O_2_ detection, consisting of a working electrode made of inexpensive SS wire with a diameter of 0.1 mm and modified with AuNPs, a counter electrode made of untreated Pt wire with a diameter of 0.1 mm, and a reference electrode made of an Ag/AgCl wire with a diameter of 0.1 mm for H_2_O_2_ was developed by Huo et al.^59^ The fabricated microsensor was applied for in vivo monitoring of H_2_O_2_ in the vein of a tomato leaf exposed to high salinity. These findings demonstrated that high salinity could stimulate H_2_O_2_ formation within 2 h. According to this study, the disposable electrochemical microsensor is suitable for continuous in vivo sensing of H_2_O_2_ in small plant tissues with minimal plant damage (Figure 7A). The applied potential of −0.6 V may lead to interference from redox active species present in the plant matrix. Lima et al.^60^ used separate microsensors for in situ monitoring of H_2_O_2_ and pH. H_2_O_2_ was determined by CV using a dual-function Pt disc microelectrode with an external silver-epoxy coating serving as the pseudoreference electrode, whereas pH was measured potentiometrically using an IrOx-coated gold microelectrode. A silver wire served as the quasi-reference electrode for both series of measurements. H_2_O_2_ was generated in *Agave tequilana* leaves after inoculation with *Enterobacter cloacae*, an endophytic bacterium. To this end, the upper epidermis of *A. tequilana* leaves was punctured with a needle to produce a tiny chamber into which a fabricated electrochemical sensor was manually inserted using a 3D micropositioner. 3,3′-Diaminobenzidine staining was used to detect H_2_O_2_ in root hairs after bacterial inoculation to assess the sensitivity of microsensor. Lower pH values were detected in *A. tequilana* leaves treated with bacteria during in situ pH measurements using a gold disc microelectrode coated with iridium oxide (IrOx), suggesting that the plant produces acidic compounds through the synthesis of secondary metabolites (Figure 7B). This microsensor could be a valuable tool for future research to understand the process of H_2_O_2_ production during the interaction between plants and endophytes. Control experiments were performed to evaluate the influence of microsensor-induced wounding on the electrochemical response and ensure the reliability of the in vivo measurements. Xu et al. used Pt-based microbiosensors modified with platinum microparticles (Pt-MPs) and poly­(*o*-phenylenediamine) (PoPD) to monitor H_2_O_2_-associated oxidative bursts in oilseed rape. In the *Sclerotinia sclerotiorum* study, the Pt/Pt-MP/PoPD working electrode was inserted into the leaf vein, and different temporal response profiles were observed in the susceptible cultivar 84039 and the resistant GOx-transgenic line GO016.^61^ In a separate study, a PoPD/Pt-MP/Pt microbiosensor inserted into the leafstalk recorded two oxidative bursts at approximately 2 and 25 h following ultraviolet A (UV-A) and ultraviolet C (UV–C) exposure.^64^

**Figure 7:**
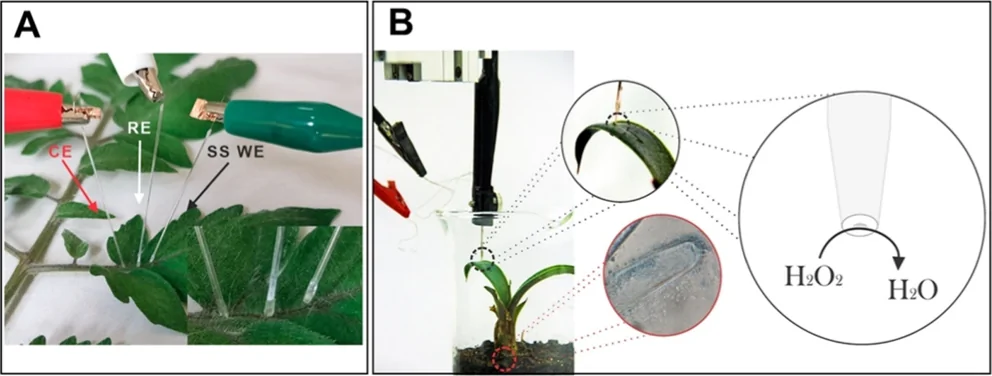
(A) An SS wire electrodeposited with AuNPs, together with a Pt wire counter electrode and an Ag/AgCl wire reference electrode, used for continuous in vivo electrochemical monitoring of H_2_O_2_ in a tomato leaf vein, Reproduced from ref 59 Available under a CC BY 4.0. license. Copyright 2022 Huo et al.; (B) A dual-function Pt disk microelectrode was prepared by inserting a Pt wire into a glass capillary and coating the capillary body with silver epoxy to serve as a pseudoreference electrode, enabling in vivo detection of H_2_O_2_ in *Agave tequilana* leaves using a single sensor, Reproduced with permission from.^60^ Copyright 2017 Elsevier B.V.

Compared with more stable analytes, ROS and RNS are highly dynamic and short-lived, making their detection inherently challenging. Electrochemical microsensors, particularly amperometric methods, are well suited for recording these transient oxidative events, especially by monitoring relatively stable species such as H_2_O_2_. However, limited selectivity due to overlapping redox responses and signal instability in complex plant matrices remains a significant challenge. Despite these limitations, these microsensors allow for direct, real-time, and localized measurements and are therefore powerful tools for in vivo and in situ monitoring oxidative signaling and stress-related redox dynamics in plants.

### 2.6 Foreign DNA Isolation

A significant concern in agriculture worldwide is the control of plant diseases caused by viruses, bacteria, fungi, and other plant pathogens.^141,142^ Over the past few decades, the geographic distribution of plant pathogens has changed significantly in response to multiple factors, including climate change.^143^ Many pathogens are now found in previously unanticipated geographic locations.^144,145^ Moreover, owing to the rise in global trade and travel, emerging and reemerging plant diseases have dispersed rapidly throughout the planet.^146,147^ As a result, growers today face a greater threat from invasive plant pathogens than before. To lessen the threat posed by plant pathogens, several advanced pathogen detection techniques have been developed, including molecular methods such as nucleic acid amplification (NAA),^145,148^ imaging and spectroscopic methods,^149,150^ nanomaterial-based volatile organic compound (VOC) sensors,^150–152^ and ELISA.^153,154^ Because they provide specific genetic information about disease-causing pathogens, NAA-based methods are among the most frequently used methods for plant pathogen identification. NAA is a labor-intensive, multistep technique in laboratory settings.^145^ Laboratory NAA methods involve sample shipment and in-house analysis, resulting in relatively long turnaround times of several days despite their high detection sensitivity and specificity.^153^ Since sample preparation remains one of the most significant barriers to the on-site molecular detection of plant pathogens, plant DNA extraction is restricted to well-equipped laboratories. A simple method for harvesting DNA from plant leaf tissues has been developed to circumvent this obstacle. Within a minute, plant or pathogenic DNA from different plant species can be isolated using an MN patch constructed from poly­(vinyl alcohol) (PVA).^69,155^ The polymeric MN patch enters plant leaf tissues during DNA extraction and breaches rigid plant cell walls to isolate intracellular DNA. The isolated DNA can be amplified by polymerase chain reaction (PCR) without additional purification. The DNA of *Phytophthora infestans* was successfully isolated from infected tomato leaves using this minimally invasive technique. The MN patch might also be used to directly isolate DNA from other plant pathogens. As a result, it has excellent potential to be developed into a rapid, on-site sample-preparation method for plant pathogen identification (Figure 8A).^155^ Paul et al.^68^ also presented a sample-to-answer (30 min) multiplexed plant pathogen diagnostic method using an integrated MN-smartphone NAA platform comprising two main parts: (I) 3D-printed smartphone imaging device for loop-mediated isothermal amplification (LAMP) reactions and detection, and (II) polymeric MN patches for rapid nucleic acid extraction within a minute (Figure 8B). The results revealed that even a needle tip from the MN patch could extract enough ribonucleic acid (RNA) for reverse transcription polymerase chain reaction (RT–PCR) or reverse-transcription loop-mediated isothermal amplification (RT–LAMP) analysis. The RNA samples extracted using MNs remained stable on their surface for up to 3 days. Similar to a benchtop thermal cycler, the MN-smartphone platform was used to detect *P. infestans* DNA and tomato spotted wilt virus (TSWV) RNA at 1 pg. Finally, multiplexed detection of *P. infestans* and TSWV was achieved using a single extraction from infected tomato leaves and amplification on a smartphone without benchtop equipment.

**Figure 8:**
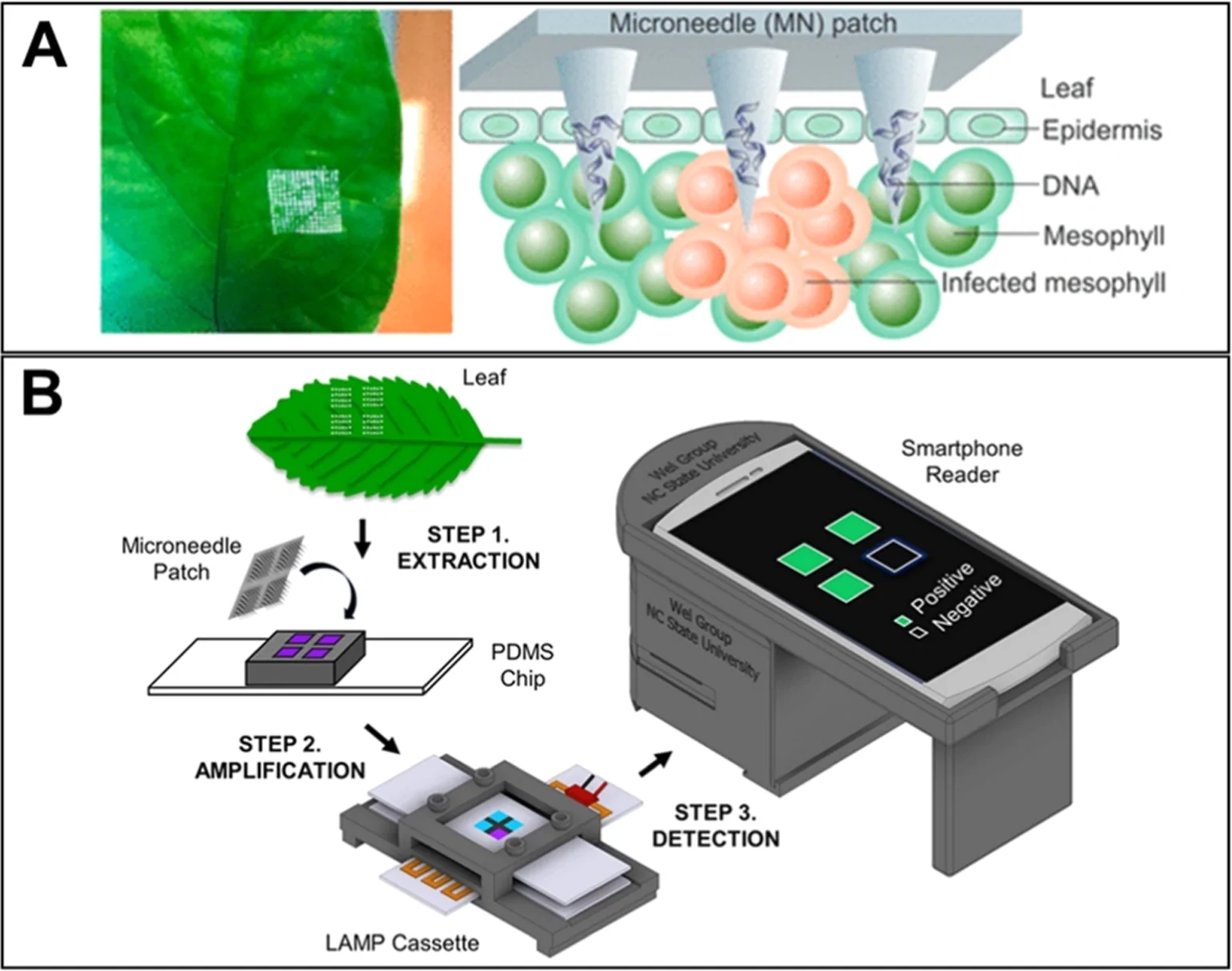
(A) Extraction of plant DNA using MN patches, Reproduced from.^155^ Copyright 2019 American Chemical Society; (B) In-field plant disease monitoring using an MN-based NAA platform integrated with a smartphone, Reproduced with permission from.^68^ Copyright 2021 Elsevier B.V.

Unlike electrochemical microsensors that directly convert biochemical or redox-active analytes into measurable electrical signals, plant pathogen detection relies on the identification of specific molecular markers, mainly nucleic acids, to achieve high specificity at the genomic level. In this regard, MN-based platforms represent a significant advance by enabling rapid and minimally invasive extraction of pathogen DNA or RNA directly from plant tissues, facilitating early and accurate detection of plant infections. When integrated with portable amplification systems, such as smartphone-based LAMP, these approaches enable rapid, in situ pathogen detection without the need for laboratory equipment. However, unlike electrochemical microsensors, these methods cannot provide continuous, real-time monitoring of plant physiological processes. Hence, MN-based molecular extraction and electrochemical microsensing can be considered as complementary approaches whose combination enables accurate pathogen identification and simultaneous monitoring of plant physiological status for comprehensive plant health assessment.

## 3 Assessment of the Measurement Accuracy

The strategy employed for quantifying analytes in plant samples using in situ and in vivo measurements with insertable microsensors is critical. A simple approach involves applying a calibration equation derived from standard solutions to calculate analyte concentrations in plant samples.^35,52^ However, this method does not account for matrix effects arising from the complex composition of plant tissues because the calibration is based on noncomplex standard solutions.

To overcome this limitation, results obtained from in vivo microsensor measurements should be validated using established reference analytical methods. These methods include techniques such as inductively coupled plasma with optical emission spectroscopy or mass spectrometry (ICP-OES or ICP–MS), atomic absorption spectroscopy (AAS),^37^ and ion chromatography^36^ for ionic species, as well as HPLC-MS or UPLC-MS for organic compounds.^48^

Accuracy can be assessed by preparing plant samples containing varying levels of the target analyte, which are then measured using the insertable microsensor. This can be achieved by cultivating plants in media with different analyte concentrations,^38^ immersing plant samples in artificial pond water with controlled analyte levels for a defined period,^38^ exposing plants to different stress conditions that induce changes in analyte concentration within the plant tissues,^48^ or by spraying the analyte solution on the plant sample.^37^ Such strategies enable comparison between expected and measured analyte concentrations, thereby supporting method validation.

An alternative strategy involves first evaluating the microsensor’s performance in solutions that mimic the plant matrix, followed by its application to in vivo measurements. For example, the standard addition method can be applied to a plant extract, homogenate,^48^ puree,^52^ or juice^44^ samples spiked with known concentrations of the analyte. Recovery rates close to 100% indicate good accuracy and the reliability of the microsensor for in vivo analysis. In summary, ensuring accurate quantification requires a comprehensive validation strategy. This should include calibration with standard solutions and following method validation, standard-addition experiments in matrix-matched samples (preferably plant extracts) to calculate recovery rates, and, importantly, confirmation through complementary reference analytical methods.

## 4 Sensor-Induced Stress

### 4.1 Sensor-Induced Wounding and Biocompatibility

Unlike surface sensors, insertable microelectrodes must penetrate the epidermis to gain direct access to plant sap.^25^ Although this penetration usually causes minimal damage, even such limited damage can activate a wound response.^25,156^ Consequently, responses induced by sensor insertion must be considered when interpreting in vivo measurement data.^157^ Although some studies have shown that plants can maintain their normal growth after microsensor insertion, biocompatibility evaluation during in-planta studies has often been limited to examining wound healing and general physiological changes, while issues such as cytotoxicity, tissue damage, and long-term stress responses have not yet been fully investigated.^25,26^ Therefore, the materials used for microsensor fabrication must possess good biocompatibility and mechanical stability, as well as high electrochemical performance.^26^ Accordingly, parameters such as diameter, length, tip angle, material, form, and insertion force are critical considerations in the development of reliable devices for plants. Furthermore, the assessment of damage caused by sensor insertion should include examining wound size and plant health indicators.^25^

One of the most important strategies to improve the mechanical biocompatibility of insertable microsensors, including MN-based sensors, is to optimize the mechanical design and reduce the mechanical mismatch between the sensor and plant tissue.^158^ For example, Kong et al.^158^ developed a PEDOT/PSS/PLGA-based MN that significantly reduced its stiffness upon electrolyte absorption. This change allowed the soft polymer network to establish better conformal contact with the plant tissue, thus enabling the recording of electrophysiological signals with fewer motion artifacts. It was shown that reducing the mechanical mismatch between the sensor and plant tissue increased the stability of the sensor–tissue interface and reduced damage compared with rigid metal electrodes. Additionally, the MN had good flexibility and showed less than 5% change in resistance at bending angles of up to 30°. Bukhamsin et al.^157^ also developed a flexible, lightweight MN sensor, demonstrating that reducing the sensor’s weight is essential to avoid triggering the thigmomorphogenetic response in the plant. Furthermore, the flexibility of the substrate allows the sensor to conform well to the leaf or stem surface and be placed on the plant without applying additional mechanical stress. In this study, the leaf was monitored for discoloration and signs of aging that could affect the measured impedance during and after the experiment. It was found that removing the sensor to examine the insertion site could affect the recorded impedance signal by causing mechanical stimulation. Moreover, the gradual closure of the epidermal wound to maintain leaf turgor pressure may affect the measured impedance values over time. However, the MN insertion site healed within 4 days, and sensor insertion did not cause permanent damage to the leaf tissue. This structure also showed greater resistance to motion artifacts and was therefore reported to be more suitable for open-air measurements. Furthermore, the authors showed that by choosing frequencies that are less susceptible to noise, the effect of electromagnetic interference can be minimized without the need for heavy shielding of the wires. These findings reveal that part of the variation recorded in in-planta measurements could result from processes associated with sensor insertion into plant tissue. Therefore, these factors should be taken into account when interpreting data from long-term monitoring.

A biocompatible hydrogel layer can be applied to MN to improve the biocompatibility of the sensor. For example, Singh et al.^62^ developed a biohydrogel-modified MN composed of Cs, rGO, and horseradish peroxidase (HRP) and demonstrated that this biohydrogel is a suitable material for fabricating bioelectrodes for plant electrochemical sensors due to its biocompatibility, hydrophilicity, porosity, and electron transport capability. Besides modifying the MN surface, changing its structural design has also been proposed as an alternative solution. Moreover, HMN can be used as protective housing for electrodes, representing another design solution. This structure reduces the risk of electrode damage during insertion into hard tissues and enables calibration, fabrication, and independent electrode maintenance contributing to continuous and reliable monitoring in the plant.^25^

Overall, existing studies show that long-term and reliable monitoring in plants depends not only on improving sensor electrochemical performance but also on optimizing mechanical design, selecting biocompatible materials, reducing the mechanical mismatch between the sensor and tissue, and accurately assessing the wound-healing response. However, biocompatibility assessment in many studies remains primarily limited to examining superficial wound healing, and further studies are needed to investigate cellular responses, tissue damage, and long-term stress responses.

### 4.2 Long-Term Stability of Implantable Microsensors

One of the most important advantages of implantable sensors is their ability to continuously monitor physiological processes in vivo. However, achieving stable performance over long periods remains a major challenge. Recent reviews of implantable electrochemical microsensors emphasize that although these sensors provide continuous and minimally invasive measurement capabilities, several issues must be addressed for long-term use, including surface biofouling, degradation of sensitive materials, and loss of specificity and measurement reliability.^159,160^

The factors affecting the performance degradation of implantable sensors can be divided into two general groups: (i) biological processes, such as membrane biofouling, biorecognition element inactivation, passivation, and fiber encapsulation, and (ii) nonbiological processes, including delamination of insulating layers, electrode corrosion and detachment, and electrical short circuits, which ultimately lead to decreased sensor performance.^159^ Among these factors, biofouling is a major limitation for long-term performance. Biofouling is the spontaneous accumulation of macromolecules or microorganisms (e.g., proteins, cells, and bacteria) that can physically restrict the diffusion of the target analyte to the sensor surface. Accumulated proteins can further stimulate the foreign body response, leading to gradual encapsulation of the sensor, blocking access to the analyte, and ultimately causing sensor failure. Hence, reducing biofouling has been proposed as a prerequisite for increasing the lifetime of implantable sensors.^159^ Although these mechanisms are well recognized in the broader implantable-sensor literature, their specific contribution to signal degradation during long-term in-planta monitoring remains insufficiently understood.

Beyond preventing biofouling and surface contamination, long-term stability during monitoring also depends on minimizing the degradation or inactivation of biosensitive materials, appropriate selection of substrate and electrode materials, balancing electrical conductivity and biocompatibility, and using antifouling coatings.^160^

#### 4.2.1 Antifouling Membranes, Protective Coatings, and Passive Diffusion Barriers

Antifouling membranes and coatings have been developed to reduce or delay biofouling on the surfaces of implantable sensors. Antifouling strategies are divided into two general groups: passive and active. Passive strategies focus on preventing the entry or binding of foulants to the sensor surface, while active strategies remove contaminants that have already absorbed onto the surface.^159^

Passive strategies have utilized natural materials, superhydrophobic surfaces, hydrophilic materials, and drug-releasing materials. The mechanisms of these strategies include creating surfaces with unfavorable thermodynamic conditions for contaminant binding, facilitating protein dissociation, mimicking natural biological systems, and modulating host responses. In contrast, active strategies primarily rely on mechanical stimulation, acoustic waves, surfactant-repelling surfaces, and temperature- or pH-sensitive materials to remove or mitigate fouling after implantation.^159^

Despite significant progress in developing antifouling strategies, many existing studies have demonstrated their feasibility only in vitro, and comprehensive evaluation of their performance in chronic in vivo studies remains limited. Therefore, it is essential to investigate the long-term performance of these strategies under in vivo conditions to develop implantable sensors with continuous and reliable monitoring capabilities.^159^

In addition to antifouling coatings, polymeric membranes have been used in some implantable electrochemical sensors. For example, Wang et al.^25^ reported that in MN sensors with an outer sensitive layer, polyurethane (PU) membrane serves as a protective barrier, provides mechanical support for the sensor, and facilitates insertion into plant tissues. In potentiometric microsensors, ion-selective membranes composed of ionophores and ion exchangers, and ion-to-electron transduction layers in SC-ISEs, enable the selective binding and transport of target ions. Furthermore, a poly­(vinyl butyral) (PVB) membrane was placed on the reference electrode of MN pH sensor to ensure potential stability regardless of the composition of the plant sap.^26^ However, the polymeric membranes used in these studies were intended to provide protection, mechanical support, ion selectivity, or reference-electrode stabilization rather than to serve as deliberately engineered passive diffusion-limiting layers for long-term monitoring.^25,26^

#### 4.2.2 Potential Self-Calibration Mechanisms

One of the main challenges in long-term monitoring is the gradual change in sensor response caused by sensitivity degradation, baseline drift, and intersensor variability. For this reason, sensors are usually calibrated by the user before or after each measurement. However, manual calibration is not suitable for repeated or continuous measurements and is not practical for in situ systems that must operate automatically in the field. Therefore, performing automatic calibration at the measurement site can reduce the need for user intervention and facilitate long-term measurements.^161^

One reported approach involves the integration of a multiplexed SC-ISE system into a microfluidic flow cell, which enables automated two-point calibration via the sequential delivery of calibration solutions. The system maintained its performance for at least 3 weeks and was used to measure K^+^ and NO_3_^–^ ions in maize plant sap samples.^161^ Although this platform was not an insertable in-planta system, translating such a calibration strategy into MN form for continuous *in-planta* monitoring remains unresolved.

Despite its importance, developing solutions that maintain measurement accuracy without frequent calibration remains a major challenge for plant MN sensors. The development of calibration-free strategies has been proposed as a major requirement for achieving long-term monitoring and commercialization this technology.^25^

In summary, existing studies indicate a need for further development in material selection, sensor design, and calibration techniques to enable long-term monitoring and facilitate the use of implantable sensors.^25,159,160^

## 5 Remote Electrochemical Monitoring of Plant Stress

Remote monitoring of plant physiological status is increasingly being pursued through integrated sensing platforms in which signal acquisition, transmission, and user-interface readout are designed together rather than treated as fully separate downstream steps.^162,163^ In parallel, a range of sensing strategies, including plant microelectrodes, wearable sensors, and emerging MN-based devices, have demonstrated the feasibility of operating microscale sensors directly on or within living plant tissues for in vivo and, in some cases, continuous monitoring.^164^ As illustrated in Figure 9, the insertable microsensor (A) serves as the primary sensing interface operating directly within plant tissue, where biochemical events are converted into electrochemical signals that initiate the monitoring pathway. Although depicted schematically as a system component, the microsensor (A) should be understood as an implanted electrochemical interface that directly interacts with the plant microenvironment and plays a central role in determining the quality and reliability of the acquired signal.^165^ This interpretation is consistent with prior in vivo studies in which electrochemical sensors and microelectrodes have been inserted into plant tissues to directly detect target analytes within the biological matrix, including glucose and variations in plant-sap solute content. In such systems, the analytical sequence begins at the implanted sensor, where target recognition generates a current or potential change; this signal is then acquired and processed by the electronic unit before being transmitted to an external device. Thus, the communication module is not the defining feature of remote monitoring; rather, it represents a continuation of the sensing pathway initiated at the microsensor interface.

**Figure 9:**
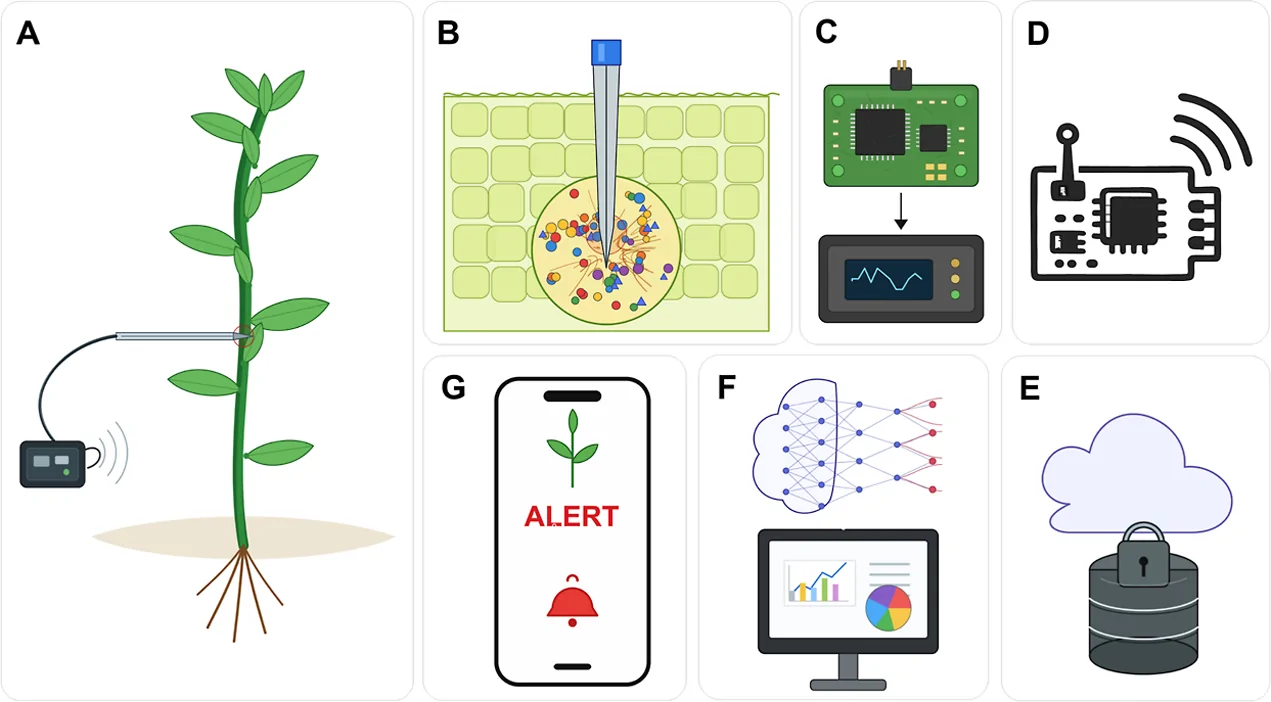
Schematic representation of a remote plant-monitoring platform based on insertable electrochemical microsensors. Following implantation into plant tissue (A), the insertable electrochemical microsensor continuously monitors the local biochemical environment. At the sensing interface, target biomarkers produce electrochemical responses (B) that are captured and prepared for digital processing by a portable electronic device (C). Communication with remote devices is established (D), allowing recorded information to be retained for later use (E). The collected information helps reveal how the plant is changing (F). This information can then be used to support everyday decisions in crop management (G).

The integration of these components is nontrivial. Plant tissues are chemically heterogeneous, and local changes in pH, ionic strength, redox-active metabolites, and fluid composition can significantly influence the electrochemical response under in vivo conditions.^54,165,166^ In addition, plant growth, tissue movement, and environmental perturbations can mechanically disturb the implanted interface, affecting positional stability and signal reproducibility.^54,165^ These observations suggest that remote monitoring systems for plants should not be directly translated from generic IoT platforms; instead, they need to be designed around the behavior of the implanted electrochemical interface itself.^162,165^

A similar conclusion emerges from studies on long-term electrochemical sensing.^167^ Continuous electrochemical monitoring is often challenged by weak signal responses, biological noise, interfacial fouling, and signal drift over time.^167,168^ Although much of this literature originates from biomedical sensing, the same underlying principles are broadly applicable here, as insertable plant microsensors operate in similarly complex biological environments. For plant applications, this implies that stable remote monitoring requires not only signal transmission but also appropriate signal conditioning, drift management, and strategies for preserving analytical performance during prolonged implantation.^165,167^

Evidence for functional integration comes from studies combining sensing, electronics, and readout within a single platform.^54,129^ Paul et al.^68^ reported an integrated MN–smartphone NAA platform for in-field diagnosis of plant diseases, combining sample acquisition, molecular analysis, and smartphone-based readout into a single portable “sample-to-answer” workflow. Although these systems target different analytes and rely on distinct transduction mechanisms, they collectively highlight the benefits of integrating the sensing interface with the downstream digital platform. This literature also helps define the appropriate role of data analysis. Modern biosensing systems increasingly rely on signal processing, filtering, and data analysis approaches to extract reliable information from continuous sensor outputs, particularly when signals are affected by noise and drift over time.^169^ In the context of insertable plant microsensors, such approaches should be considered practical tools for improving the interpretation of long-term in vivo measurements. To predict future time-series data, Zhai et al.^83^ applied a nonlinear autoregressive neural network (NARNN) to data acquired over 1 h using an implanted MN K^+^-selective electrode in lettuce. The model used delayed values of the recorded signal as feedback inputs to learn its nonlinear temporal behavior and predict subsequent K^+^ values, with a feedback delay of 20 s. The NARNN captured the transition from the relatively large fluctuations observed after sensor insertion to a more stable signal response. Applying an Equiripple low-pass filter reduced signal noise and improved model performance, as reflected by lower mean absolute error (MAE) and root-mean-square error (RMSE) values and a higher coefficient of determination (R^2^). Although wireless transmission and remote access were not included, the study illustrates how predictive analysis can be used to interpret continuous signals from insertable plant sensors.

Energy supply should also be considered from this integrated perspective. Long-term field deployment requires more than simple connectivity it typically demands low-power operation or energy-aware system design capable of sustaining sensing, processing, and transmission over extended periods.^169^ For this reason, a compelling future direction is not a plant sensor attached to a generic remote system, but a codesigned platform in which electrochemical sensing, electronics, data handling, and communication are developed together.

Overall, the available literature suggests that a key advance in this area lies not in discussing implantable microsensors and remote monitoring as separate themes but in understanding how one enables the other. From this perspective, insertable electrochemical microsensors can be viewed as the analytical core of remote monitoring systems, with signal acquisition, data processing, and communication structured around the requirements of the in vivo plant interface.

## 6 Challenges and Future Perspectives

Microsensors are widely used across industries to monitor various physicochemical parameters, including pressure, temperature, and humidity. This review highlights the potential of microsensors in agriculture to provide timely information on plant health status. However, several challenges must be addressed before insertable microsensors can be widely adopted in smart agriculture, including material biocompatibility and mechanical stability, surface fouling, spatial heterogeneity, in vivo measurement accuracy, data management, and sustainable power supply, as summarized in Figure 10. The materials used in the fabrication of microsensors are of great importance, as they must possess good mechanical stability and excellent biocompatibility to avoid cytotoxicity in biological tissues during long-term insertion. In fact, the wounding caused by the penetration of microsensors into plant tissues can trigger the plant’s defense mechanisms. Advances in materials science may help address this problem by designing appropriate composite materials that simultaneously possess biocompatibility, mechanical stability, and electrical conductivity. Furthermore, proper and effective insertion of microsensors into plants without causing damage can be achieved by designing a robotic micropositioner that reproducibly inserts them with controlled force.

**Figure 10:**
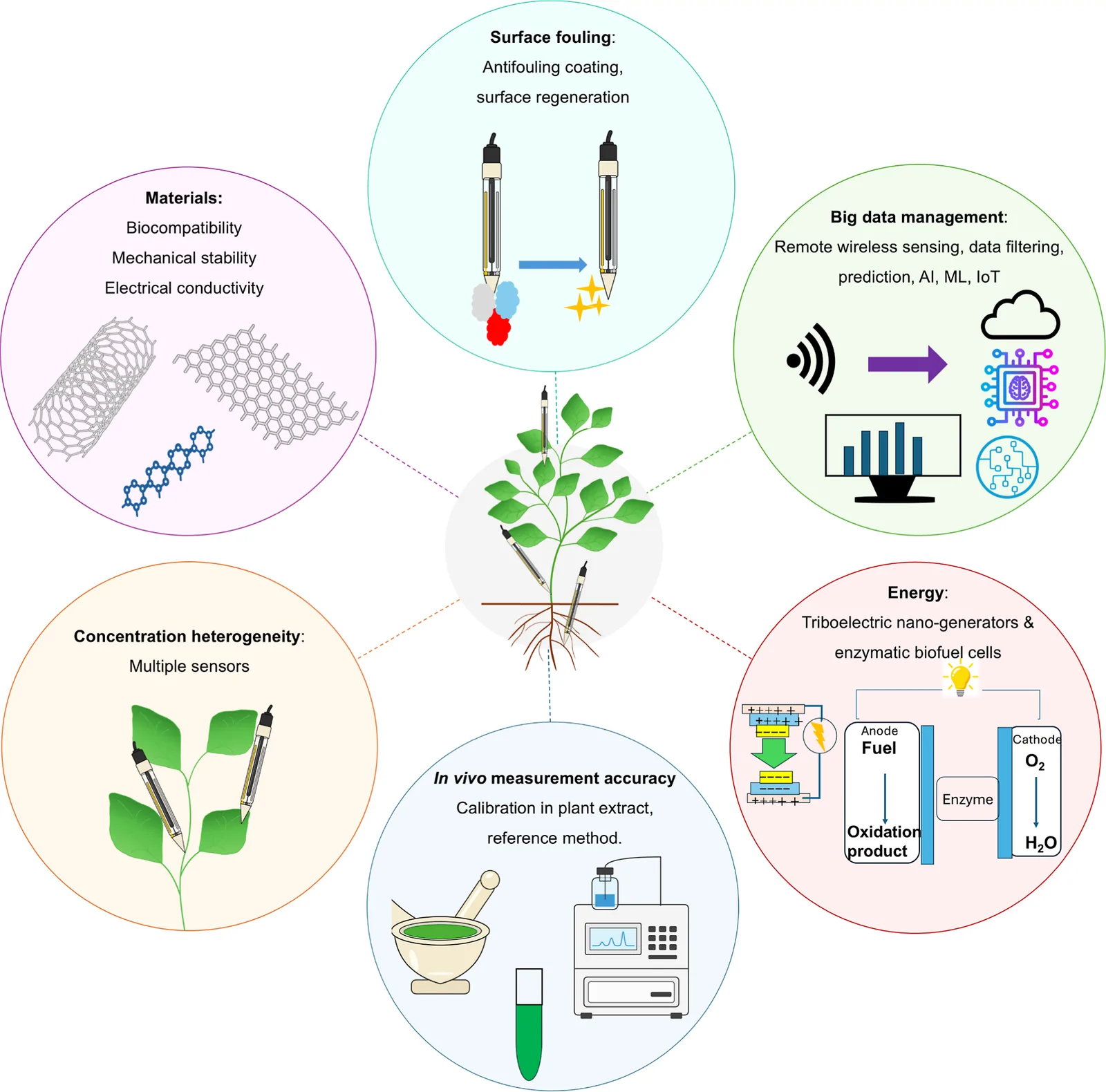
Key challenges and future directions for insertable plant microsensors.

Moreover, the concentration of stress biomarkers is not uniformly distributed throughout the plant. This necessitates the use of multiple microsensors in different parts of the plant to determine the spatial distribution of the biomarker. The information obtained from microelectrodes can be complemented by imaging technologies, such as fluorescence microscopy, to provide more in-depth and detailed information about how plants respond to stressors.

Additionally, determining the concentration dependence of the electrochemical signal inside the plant is complicated. Most often, the calibration equation is obtained ex situ and used to calculate the concentration inside the plant. The accuracy of this approach is limited by the complex plant tissue matrix. Accuracy is further affected by abrupt changes in pH and ionic strength resulting from the release of stress biomarkers. The high viscosity of plant’s fluids may limit the diffusion of the target analyte to the electrode, increasing the response time. Plant metabolites, proteins, or other biomolecules can accumulate on the microelectrode surface. This gradually changes the electrochemical interface, reducing sensitivity. The use of antifouling coatings, self-cleaning nanostructured surfaces, electrochemical regeneration techniques, and surface functionalization with biocompatible polymers can minimize nonspecific adsorption and help maintain long-term accuracy. Alternatively, the mass production of low-cost disposable microsensors provides a practical solution by eliminating the need for long-term maintenance and surface regeneration. Additionally, the presence of various redox-active biological compounds within the plant’s tissue may limit selectivity. Although the use of biological receptors can improve selectivity, harsh environmental conditions can degrade these bioreceptors. The development of protective coatings can help sensors remain unaffected and be protected from environmental conditions.

The use of microsensors for continuous monitoring of plant stress biomarkers over long periods requires an intelligent system characterized by accurate sensing in a short time, efficient transfer of the collected data, and low energy consumption. Continuous measurements generate very large data sets, which pose challenges for data handling and interpretation. Noise filtering, secure storage, and the conversion of raw signals into useful information require advanced computational capabilities. Integrating advances in electronics and microelectronics into the sensing platform aims to leverage their specific capabilities for agricultural applications. To this end, the application of IoT,^170^ an advanced technological innovation, enables wireless operation and communication services that facilitate data collection and transmission via satellite networks, thereby promoting remote monitoring. Furthermore, technological advances enable the development of miniaturized and portable sensing systems, which are essential for modern sensing applications. Indeed, electronic products such as smartphones and Bluetooth devices are used for their capabilities in measurement, data collection, and transmission. Interestingly, smartphones can function as electrochemical analyzers, providing the portability required for point-of-need detection. Additionally, web browsers provide an interface for sending and receiving acquired data with no time delay. Moreover, automated data analysis and the rational use of AI and ML in data processing enable the simple and rapid interpretation of complex physiological changes. Therefore, data obtained from insertable microelectrodes can be analyzed using ML algorithms, enabling effective modeling for predicting plant stress responses.^83^

Another challenge is the reliable power supply required for remote and continuous monitoring systems. Since these systems must operate for long periods under field conditions, low-power circuit design and autonomous energy technologies are of great importance. Therefore, energy-harvesting solutions, including triboelectric nanogenerators and other self-powered systems, could play an important role in the future development of plant microsensors.^171^ Alternatively, enzymatic biofuel cells (EBFCs) represent another option for energy harvesting in plant microsensor systems. In these systems, glucose is enzymatically oxidized at the bioanode, and O_2_ is reduced at the biocathode. This process drives electron transfer through an external circuit and generates electrical power. A membraneless glucose–O_2_ biofuel cell was shown to generate electrical power after being implanted in a living grape. Notably, for 1 week operation, the dimensions of such a cell could be smaller than those of a battery, highlighting its potential for miniaturizing the energy source in low-power systems.^172^ Similarly, enzyme-modified electrodes implanted in a living plant responded to visible light and harvested glucose and O_2_ produced during photosynthesis to generate electrical power.^173^ However, the use of EBFCs as power sources for insertable plant microsensors is still at the developmental stage. Wired EBFCs have shown limited current density and operational lifetime, and it has recently been reported that EBFCs still face challenges related to low power output and power density, enzyme stability and activity, operational lifetime, miniaturization, and integration into biosensing systems, which must be addressed in the future.^172,174^

Another option involves the use of passive or battery-free radio frequency identification (RFID) sensors. In backscatter-based RFID systems, the reader transmits an electromagnetic wave to the RFID tag, where the received energy is converted into DC power to activate the chip, while information is transmitted through changes in the antenna load and reflection of the carrier wave.^175,176^ This technology has been investigated for smart agriculture, soil moisture monitoring, and leaf moisture detection. However, existing studies have mainly focused on RFID systems for environmental, soil, or leaf monitoring rather than on the direct integration of RFID with implantable electrochemical microsensors. Therefore, the use of RFID in such systems still requires more careful design to address communication distance, readout stability, and compatibility among the RFID tag, readout circuit, and sensor–plant interface.

Moreover, insertable microelectrodes can be used to remotely map plant leaves, enabling a real-time, on-the-spot understanding of plant physiological changes.

Overcoming these problems will be required to advance insertable microsensors from the laboratory to the farms as effective precision agriculture tools. By combining advances in materials, smart power technologies, and sophisticated data analytics, these systems can become scalable, reliable, and environmentally sustainable technologies for future agriculture.

## 7 Conclusion

Insertable and implantable microsensors have enabled direct, online monitoring of plant physiological processes and opened new horizons for the dynamic in vivo assessment of stress biomarkers. Advances in functional materials, microsensor design, signal transduction strategies, and communication systems have expanded the scope of this technology from the measurement of a single analyte to the monitoring of a diverse range of biomarkers. As a result, this technology has become a suitable platform for developing a new generation of plant monitoring systems.

Despite these advances, the commercialization and widespread application of this technology still face significant obstacles. Long-term performance stability, biocompatibility, reduction of the mechanical mismatch between the microsensor and plant tissue, resistance to biofouling, maintenance of selectivity and sensitivity, development of reliable calibration strategies, and integration of data transmission and processing systems remain the major challenges facing microsensors. Furthermore, most of the systems introduced so far have been evaluated at the laboratory scale or in proof-of-concept studies, and there is limited evidence of their long-term performance under real field conditions.

The future development of this field requires the simultaneous design of sensor materials, microsensor architecture, electronic systems, self-powered technologies, wireless communication systems, and advanced data processing methods. Moving toward multianalyte, low-power, and integrated systems, along with evaluating their performance under real conditions, can reduce the gap between laboratory prototypes and practical applications. Achieving these goals will pave the way for the development of reliable plant-monitoring systems and the wider use of plant-insertable microsensors in smart agriculture.

## Abbreviations

^1^O_2_: singlet oxygen
AAS: atomic absorption spectroscopy
ABA: abscisic acid
AgNPs: silver nanoparticles
AI: artificial intelligence
AIB: α-aminoisobutyric acid
AMP: adenosine monophosphate
ATP: adenosine triphosphate
AuNPs: gold nanoparticles
BR: brassinosteroids
CA: chlorogenic acid
ChemFET: chemically sensitive field-effect transistor
ChA: chronoamperometry
CK: cytokinins
CNC: cellulose nanocrystals
CNT: carbon nanotube
CO_2_: carbon dioxide
Cs: chitosan
CV: cyclic voltammetry
DBS: dibutyl sebacate
DNA: deoxyribonucleic acid
DPV: differential pulse voltammetry
EBFCs: enzymatic biofuel cells
ET: ethylene
EIS: electrochemical impedance spectroscopy
ELISA: enzyme-linked immunosorbent assay
ERGO: electrochemically reduced graphene oxide
f-MWCNTs: functionalized multiwalled carbon nanotubes
FET: field-effect transistor
GA: gallic acid
GAs: gibberellins
GCE: glassy carbon electrode
GelMA: methacrylamidated gelatin
GO: graphene oxide
GOGAT: glutamate synthase
GOPS: 3-(glycidyloxypropyl)­trimethoxysilane
GOx: glucose oxidase
G: graphene
GrRE: graphite rod electrode
GS: glutamine synthetase
H_2_O_2_: hydrogen peroxide
HMNA: hollow microneedle array
HO^•^: hydroxyl radical
HOCl/OCl^–^: hypochlorous acid/hypochlorite
HPLC: high-performance liquid chromatography
HPTLC: high-performance thin-layer chromatography
HRP: horseradish peroxidase
HXKs: hexokinases
IAA: indole-3-acetic acid
ICP-MS: inductively coupled plasma mass spectrometry
ICP-OES: inductively coupled plasma optical emission spectroscopy
IDME: interdigitated microelectrode
IoT: Internet of Things
IRGAs: infrared gas analyzers
IrOx: iridium oxide
ISMEs: ion-selective microelectrodes
JA: jasmonates
KTCPB: potassium tetrakis­(4-chlorophenyl)­borate
l-Trp: L-tryptophan
LIG: laser-induced graphene
LIGS: laser-induced graphene sheets
MAE: mean absolute error
MIP: molecularly imprinted polymer
ML: machine learning
MN: microneedle
MNA: microneedle array
MPt: micromachined platinum microelectrode
MS: mass spectrometry
MTDDA·NO_3_: methyltridodecylammonium nitrate
MWCNTs: multiwalled carbon nanotubes
MWCNTs–COOH: carboxylated multiwalled carbon nanotubes
MXene: two-dimensional transition-metal carbide, nitride, or carbonitride
N-CNTs: nitrogen-doped carbon nanotubes
NAA: nucleic acid amplification
NADPH: nicotinamide adenine dinucleotide phosphate
NARNN: nonlinear autoregressive neural network
NDG: nitrogen-doped graphene
NiR: nitrite reductase
^•^NO: nitric oxide
^•^NO_2_: nitrogen dioxide
NPPt: nanoporous platinum
NR: nitrate reductase
NSCCs: nonselective cation channels
O_2_: oxygen
O_2_^•–^: superoxide
OECT: organic electrochemical transistor
ONOO^–^: peroxynitrite
PANI: polyaniline
PB: Prussian blue
pCa: negative base-10 logarithm of Ca^2+^ activity
PCR: polymerase chain reaction
PDA: polydopamine
PDMS: poly­(dimethylsiloxane)
PEDOT/PSS: poly­(3,4-ethylenedioxythiophene):poly­(styrenesulfonate)
PET: polyethylene terephthalate
PI: polyimide
PoPD: poly­(*o*-phenylenediamine)
PSA: poly­(sulfosalicylic acid)
PST: poly­(safranine T)
Pt: platinum
Pt-MPs: platinum microparticles
PtNPs: platinum nanoparticles
PU: polyurethane
PVA: poly­(vinyl alcohol)
PVB: poly­(vinyl butyral)
PVC: poly­(vinyl chloride)
QANFAs: quasi-aligned nanofiber arrays
RFID: radio frequency identification
rGO: reduced graphene oxide
RMSE: root-mean-square error
RNA: ribonucleic acid
RNS: reactive nitrogen species
ROO^•^: peroxyl radical
ROS: reactive oxygen species
RT-LAMP: reverse-transcription loop-mediated isothermal amplification
RT–PCR: reverse transcription polymerase chain reaction
SA: salicylic acid
SC-ISEs: solid-contact ion-selective electrodes
SOD: superoxide dismutase
SPE: screen-printed electrode
SS: stainless steel
SS-MN: stainless-steel microneedle
TDN: tetrahedral DNA nanostructure
TiC@C: titanium carbide–carbon core–shell structure
TSWV: tomato spotted wilt virus
UPLC: ultraperformance liquid chromatography
UV-A: ultraviolet A
UV–B: ultraviolet B
UV–C: ultraviolet C
VB_12_: vitamin B_12_
VG: vertical graphene
VOC: volatile organic compound
